# Bivariate random-effects meta-analysis and the estimation of between-study correlation

**DOI:** 10.1186/1471-2288-7-3

**Published:** 2007-01-12

**Authors:** Richard D Riley, Keith R Abrams, Alexander J Sutton, Paul C Lambert, John R Thompson

**Affiliations:** 1 Centre for Medical Statistics and Health Evaluation, School of Health Sciences, University of Liverpool, Shelley's Cottage, Brownlow Street, Liverpool, L69 3GS, UK; 2 Centre for Biostatistics and Genetic Epidemiology, Department of Health Sciences, University of Leicester, 2nd Floor, Adrian Building, University Road, Leicester, LE1 7RH, UK

## Abstract

**Background:**

When multiple endpoints are of interest in evidence synthesis, a multivariate meta-analysis can jointly synthesise those endpoints and utilise their correlation. A multivariate random-effects meta-analysis must incorporate and estimate the between-study correlation (*ρ*_*B*_).

**Methods:**

In this paper we assess maximum likelihood estimation of a general normal model and a generalised model for bivariate random-effects meta-analysis (BRMA). We consider two applied examples, one involving a diagnostic marker and the other a surrogate outcome. These motivate a simulation study where estimation properties from BRMA are compared with those from two separate univariate random-effects meta-analyses (URMAs), the traditional approach.

**Results:**

The normal BRMA model estimates *ρ*_*B *_as -1 in both applied examples. Analytically we show this is due to the maximum likelihood estimator sensibly truncating the between-study covariance matrix on the boundary of its parameter space. Our simulations reveal this commonly occurs when the number of studies is small or the within-study variation is relatively large; it also causes upwardly biased between-study variance estimates, which are inflated to compensate for the restriction on ρ^
 MathType@MTEF@5@5@+=feaafiart1ev1aaatCvAUfKttLearuWrP9MDH5MBPbIqV92AaeXatLxBI9gBaebbnrfifHhDYfgasaacH8akY=wiFfYdH8Gipec8Eeeu0xXdbba9frFj0=OqFfea0dXdd9vqai=hGuQ8kuc9pgc9s8qqaq=dirpe0xb9q8qiLsFr0=vr0=vr0dc8meaabaqaciaacaGaaeqabaqabeGadaaakeaaiiGacuWFbpGCgaqcaaaa@2E83@_*B*_. Importantly, this does not induce any systematic bias in the pooled estimates and produces conservative standard errors and mean-square errors. Furthermore, the normal BRMA is preferable to two normal URMAs; the mean-square error and standard error of pooled estimates is generally smaller in the BRMA, especially given data missing at random. For meta-analysis of proportions we then show that a generalised BRMA model is better still. This correctly uses a binomial rather than normal distribution, and produces better estimates than the normal BRMA and also two generalised URMAs; however the model may sometimes not converge due to difficulties estimating *ρ*_*B*_.

**Conclusion:**

A BRMA model offers numerous advantages over separate univariate synthesises; this paper highlights some of these benefits in both a normal and generalised modelling framework, and examines the estimation of between-study correlation to aid practitioners.

## Background

Traditionally, meta-analysis models combine summary measures of a single quantitative endpoint, taken from different studies, to produce a single pooled result. However, multiple pooled results are required whenever there are multiple outcomes [1] or multiple treatment groups [2]. A multivariate meta-analysis model uses the correlation between the endpoints and obtains the multiple pooled results collectively [3,4]. For example, Reitsma et al. [5] have suggested a bivariate random-effects meta-analysis (BRMA) to jointly synthesise logit-sensitivity and logit-specificity values from diagnostic studies. Multivariate meta-analysis has been quite widely used, including application to genetic associations [6], surrogate endpoints [7,8], psychological findings [9] and prognostic markers [10].

Often the advantage of a multivariate random-effects meta-analysis lies in its ability to use the between-study correlation of the multiple endpoints of interest. For example, in diagnostic studies the sensitivity and specificity are usually negatively correlated across studies due to the use of different thresholds [5]. Van Houwelingen et al. [4] use the between-study correlation to describe the shape of the bivariate relationship between the true log-odds in a treatment group and the true log-odds in a control group (baseline risk). Riley et al. [10] algebraically assess BRMA and show that the correlation allows a 'borrowing of strength' across endpoints. This leads to pooled estimates that have a smaller standard error than those from corresponding univariate random-effects meta-analyses (URMAs), especially when some endpoints are missing at random across studies.

Some recent articles have indicated the between-study correlation may often be estimated at the end of its parameter space, as +1 or -1. Thompson et al. [6] apply a normal BRMA model to genetic studies of coronary heart disease and report that the between-study correlation was 'poorly estimated' with the likelihood peaking at +1, and an estimate of +1 has also been reported in other applications [4,11]. To aid practitioners, in this paper we analytically consider why this occurs, and then explore the impact, if any, this has on the pooled estimates and between-study variance estimates. This investigation also allows us to examine the general benefits of BRMA over URMA, to build on a number of other recent articles [5,10], and encourage a greater use and appreciation of BRMA in practice.

The outline of the paper is as follows. We begin by introducing the general normal model for BRMA and discuss analytically why the between-study correlation can be estimated at the edge of its parameter space. We then apply the model to two real examples from the literature, one involving a diagnostic marker and one involving a surrogate outcome, and these both give a between-study correlation estimate of -1. This then motivates a realistic simulation study to examine how the estimate of between-study correlation affects the statistical properties of the pooled and between-study variance estimates. It also allows us to compare the performance of the normal BRMA model to two separate URMAs, the more common approach in practice [10]. We then extend our work to consider meta-analysis of proportions, and highlight why a generalised model for BRMA is preferred to the general normal BRMA model [12], and also two separate generalised URMA models. We conclude by summarising the broad benefits of BRMA for practitioners, and discuss future research priorities.

## Methods

In this section we introduce a hierarchical normal framework for BRMA and URMA. We describe how the BRMA normal model is estimated, and analytically consider the estimation of between-study correlation. We then describe the rationale and methodology for our simulation study of BRMA versus URMA. To ensure a real world context, this section also includes two motivating examples from the medical literature where a BRMA is potentially important.

### Motivating example 1 – the telomerase data

Glas et al. [13] systematically review the sensitivity and specificity of tumour markers used for diagnosing primary bladder cancer. One of these markers was telomerase, a ribonucleoprotein enzyme, evaluated in 10 studies as shown in Table 1. Rather than applying a URMA independently for sensitivity and specificity, the authors jointly synthesise the logit-transformed sensitivity and the logit-transformed specificity in a normal BRMA model as described below and recently proposed elsewhere [5].

**Table 1:** The telomerase data taken from the bladder cancer review of Glas et al. [13]

**Study**	Number of patients with bladder cancer	Number of patients with bladder cancer and a positive test result	**Sensitivity**	Logit-sensitivity *Y*_*i*1_	Standard error of logit-sensitivity *S*_*i*1_	Number of patients without bladder cancer	Number of patients without bladder cancer and a negative test result	**Specificity**	Logit-specificity *Y*_*i*2_	Standard error of logit-specificity *S*_*i*2_	Within-study correlation *ρ*_*Wi*_
1	33	25	0.758	1.139	0.406	26	25	0.962	3.219	1.020	0
2	21	17	0.810	1.447	0.556	14	11	0.786	1.299	0.651	0
3	104	88	0.846	1.705	0.272	47	31	0.660	0.661	0.308	0
4	26	16	0.615	0.470	0.403	83	80	0.964	3.283	0.588	0
5	57	40	0.702	0.856	0.290	138	137	0.993	4.920	1.004	0
6	47	38	0.809	1.440	0.371	30	24	0.800	1.386	0.456	0
7*	43	23.5	0.547	0.187	0.306	13	12.5	0.962	3.219	1.442	0
8	33	27	0.818	1.504	0.451	20	18	0.900	2.197	0.745	0
9	17	14	0.824	1.540	0.636	32	29	0.906	2.269	0.606	0
10	44	37	0.841	1.665	0.412	29	7	0.241	-1.145	0.434	0

* 0.5 was added to each cell of the 2 by 2 table for this study, as a continuity correction was needed given there were zero false negatives

### A general normal model for bivariate random-effects meta-analysis (BRMA)

Suppose that *i = 1 to n *studies are identified by a systematic review, and that two endpoints (*j = 1 or 2*) are available from each study. Each study supplies summary measures, *Y*_*ij*_, and associated standard errors, *s*_*ij*_, for each endpoint. For instance, for diagnostic studies Reitsma et al. [5] suggest the logit-sensitivity is *Y*_*i*1 _and the logit-specificity is *Y*_*i*2_. Each summary statistic (*Y*_*ij*_) is assumed to be an estimate of a true value (*θ*_*ij*_) in each study, and in a hierarchical structure each *θ*_*ij *_is assumed to be drawn from a distribution with mean (or 'pooled') value *β*_*j *_and between-study variance τj2
 MathType@MTEF@5@5@+=feaafiart1ev1aaatCvAUfKttLearuWrP9MDH5MBPbIqV92AaeXatLxBI9gBaebbnrfifHhDYfgasaacH8akY=wiFfYdH8Gipec8Eeeu0xXdbba9frFj0=OqFfea0dXdd9vqai=hGuQ8kuc9pgc9s8qqaq=dirpe0xb9q8qiLsFr0=vr0=vr0dc8meaabaqaciaacaGaaeqabaqabeGadaaakeaaiiGacqWFepaDdaqhaaWcbaGaemOAaOgabaGaeGOmaidaaaaa@30F4@. If *Y*_*ij*_/*θ*_*ij *_and *θ*_*ij *_are both assumed normally distributed then the BRMA can be specified as:

(Yi1Yi2)~N((θi1θi2),δi)δi=(si12si1si2ρwisi1si2ρwisi22)(θi1θi2)~N((β1β2),Ω)Ω=(τ12τ1τ2ρBτ1τ2ρBτ22)     (1)
 MathType@MTEF@5@5@+=feaafiart1ev1aaatCvAUfKttLearuWrP9MDH5MBPbIqV92AaeXatLxBI9gBaebbnrfifHhDYfgasaacH8akY=wiFfYdH8Gipec8Eeeu0xXdbba9frFj0=OqFfea0dXdd9vqai=hGuQ8kuc9pgc9s8qqaq=dirpe0xb9q8qiLsFr0=vr0=vr0dc8meaabaqaciaacaGaaeqabaqabeGadaaakeaafaqaaeGacaaabaWaaeWaaeaafaqabeGabaaabaGaemywaK1aaSbaaSqaaiabdMgaPjabigdaXaqabaaakeaacqWGzbqwdaWgaaWcbaGaemyAaKMaeGOmaidabeaaaaaakiaawIcacaGLPaaacqGG+bGFcqWGobGtdaqadaqaamaabmaabaqbaeqabiqaaaqaaGGaciab=H7aXnaaBaaaleaacqWGPbqAcqaIXaqmaeqaaaGcbaGae8hUde3aaSbaaSqaaiabdMgaPjabikdaYaqabaaaaaGccaGLOaGaayzkaaGaeiilaWccceGae4hTdq2aaSbaaSqaaiabdMgaPbqabaaakiaawIcacaGLPaaaaeaacqGF0oazdaWgaaWcbaGaemyAaKgabeaakiabg2da9maabmaabaqbaeqabiGaaaqaaiabdohaZnaaDaaaleaacqWGPbqAcqaIXaqmaeaacqaIYaGmaaaakeaacqWGZbWCdaWgaaWcbaGaemyAaKMaeGymaedabeaakiabdohaZnaaBaaaleaacqWGPbqAcqaIYaGmaeqaaOGae8xWdi3aaSbaaSqaaiabdEha3jabdMgaPbqabaaakeaacqWGZbWCdaWgaaWcbaGaemyAaKMaeGymaedabeaakiabdohaZnaaBaaaleaacqWGPbqAcqaIYaGmaeqaaOGae8xWdi3aaSbaaSqaaiabdEha3jabdMgaPbqabaaakeaacqWGZbWCdaqhaaWcbaGaemyAaKMaeGOmaidabaGaeGOmaidaaaaaaOGaayjkaiaawMcaaaqaamaabmaabaqbaeqabiqaaaqaaiab=H7aXnaaBaaaleaacqWGPbqAcqaIXaqmaeqaaaGcbaGae8hUde3aaSbaaSqaaiabdMgaPjabikdaYaqabaaaaaGccaGLOaGaayzkaaGaeiOFa4NaemOta40aaeWaaeaadaqadaqaauaabeqaceaaaeaacqWFYoGydaWgaaWcbaGaeGymaedabeaaaOqaaiab=j7aInaaBaaaleaacqaIYaGmaeqaaaaaaOGaayjkaiaawMcaaiabcYcaSiab+L6axbGaayjkaiaawMcaaaqaaiab+L6axjabg2da9maabmaabaqbaeqabiGaaaqaaiab=r8a0naaDaaaleaacqaIXaqmaeaacqaIYaGmaaaakeaacqWFepaDdaWgaaWcbaGaeGymaedabeaakiab=r8a0naaBaaaleaacqaIYaGmaeqaaOGae8xWdi3aaSbaaSqaaiabdkeacbqabaaakeaacqWFepaDdaWgaaWcbaGaeGymaedabeaakiab=r8a0naaBaaaleaacqaIYaGmaeqaaOGae8xWdi3aaSbaaSqaaiabdkeacbqabaaakeaacqWFepaDdaqhaaWcbaGaeGOmaidabaGaeGOmaidaaaaaaOGaayjkaiaawMcaaaaacaWLjaGaaCzcamaabmaabaGaeGymaedacaGLOaGaayzkaaaaaa@A9E1@

This model is the general normal model for BRMA [4], where **δ**_*i *_and **Ω **are the within-study and the between-study covariance matrices respectively. Usually of key interest from the analysis are the pooled estimates of *β*_1 _and *β*_2_, although sometimes an estimated function of these may be desired; for instance in the telomerase example an estimate of the log of the diagnostic odds ratio is given by β^
 MathType@MTEF@5@5@+=feaafiart1ev1aaatCvAUfKttLearuWrP9MDH5MBPbIqV92AaeXatLxBI9gBaebbnrfifHhDYfgasaacH8akY=wiFfYdH8Gipec8Eeeu0xXdbba9frFj0=OqFfea0dXdd9vqai=hGuQ8kuc9pgc9s8qqaq=dirpe0xb9q8qiLsFr0=vr0=vr0dc8meaabaqaciaacaGaaeqabaqabeGadaaakeaaiiGacuWFYoGygaqcaaaa@2E64@_1 _+ β^
 MathType@MTEF@5@5@+=feaafiart1ev1aaatCvAUfKttLearuWrP9MDH5MBPbIqV92AaeXatLxBI9gBaebbnrfifHhDYfgasaacH8akY=wiFfYdH8Gipec8Eeeu0xXdbba9frFj0=OqFfea0dXdd9vqai=hGuQ8kuc9pgc9s8qqaq=dirpe0xb9q8qiLsFr0=vr0=vr0dc8meaabaqaciaacaGaaeqabaqabeGadaaakeaaiiGacuWFYoGygaqcaaaa@2E64@_2_. The BRMA differs from two independent URMAs by the inclusion of the within-study correlations (i.e. the *ρ*_*Wi *_s) and the between-study correlation (*ρ*_*B*_). Equation (2) is equivalent to two independent URMAs when *ρ*_*Wi *_= *ρ*_*B *_= 0 for all *i*, i.e. there is zero correlation:

(Yi1Yi2)~N((θi1θi2),δi)δi=(si1200si22)(θi1θi2)~N((β1β2),Ω)Ω=(τ1200τ22)     (2)
 MathType@MTEF@5@5@+=feaafiart1ev1aaatCvAUfKttLearuWrP9MDH5MBPbIqV92AaeXatLxBI9gBaebbnrfifHhDYfgasaacH8akY=wiFfYdH8Gipec8Eeeu0xXdbba9frFj0=OqFfea0dXdd9vqai=hGuQ8kuc9pgc9s8qqaq=dirpe0xb9q8qiLsFr0=vr0=vr0dc8meaabaqaciaacaGaaeqabaqabeGadaaakeaafaqaaeGacaaabaWaaeWaaeaafaqabeGabaaabaGaemywaK1aaSbaaSqaaiabdMgaPjabigdaXaqabaaakeaacqWGzbqwdaWgaaWcbaGaemyAaKMaeGOmaidabeaaaaaakiaawIcacaGLPaaacqGG+bGFcqWGobGtdaqadaqaamaabmaabaqbaeqabiqaaaqaaGGaciab=H7aXnaaBaaaleaacqWGPbqAcqaIXaqmaeqaaaGcbaGae8hUde3aaSbaaSqaaiabdMgaPjabikdaYaqabaaaaaGccaGLOaGaayzkaaGaeiilaWccceGae4hTdq2aaSbaaSqaaiabdMgaPbqabaaakiaawIcacaGLPaaaaeaacqGF0oazdaWgaaWcbaGaemyAaKgabeaakiabg2da9maabmaabaqbaeqabiGaaaqaaiabdohaZnaaDaaaleaacqWGPbqAcqaIXaqmaeaacqaIYaGmaaaakeaacqaIWaamaeaacqaIWaamaeaacqWGZbWCdaqhaaWcbaGaemyAaKMaeGOmaidabaGaeGOmaidaaaaaaOGaayjkaiaawMcaaaqaamaabmaabaqbaeqabiqaaaqaaiab=H7aXnaaBaaaleaacqWGPbqAcqaIXaqmaeqaaaGcbaGae8hUde3aaSbaaSqaaiabdMgaPjabikdaYaqabaaaaaGccaGLOaGaayzkaaGaeiOFa4NaemOta40aaeWaaeaadaqadaqaauaabeqaceaaaeaacqWFYoGydaWgaaWcbaGaeGymaedabeaaaOqaaiab=j7aInaaBaaaleaacqaIYaGmaeqaaaaaaOGaayjkaiaawMcaaiabcYcaSiab+L6axbGaayjkaiaawMcaaaqaaiab+L6axjabg2da9maabmaabaqbaeqabiGaaaqaaiab=r8a0naaDaaaleaacqaIXaqmaeaacqaIYaGmaaaakeaacqaIWaamaeaacqaIWaamaeaacqWFepaDdaqhaaWcbaGaeGOmaidabaGaeGOmaidaaaaaaOGaayjkaiaawMcaaaaacaWLjaGaaCzcamaabmaabaGaeGOmaidacaGLOaGaayzkaaaaaa@82B9@

In equation (1) it is common to assume the sij2
 MathType@MTEF@5@5@+=feaafiart1ev1aaatCvAUfKttLearuWrP9MDH5MBPbIqV92AaeXatLxBI9gBaebbnrfifHhDYfgasaacH8akY=wiFfYdH8Gipec8Eeeu0xXdbba9frFj0=OqFfea0dXdd9vqai=hGuQ8kuc9pgc9s8qqaq=dirpe0xb9q8qiLsFr0=vr0=vr0dc8meaabaqaciaacaGaaeqabaqabeGadaaakeaacqWGZbWCdaqhaaWcbaGaemyAaKMaemOAaOgabaGaeGOmaidaaaaa@31F2@ s and *ρ*_*Wi *_s are known [1,4]. Including the uncertainty of the sij2
 MathType@MTEF@5@5@+=feaafiart1ev1aaatCvAUfKttLearuWrP9MDH5MBPbIqV92AaeXatLxBI9gBaebbnrfifHhDYfgasaacH8akY=wiFfYdH8Gipec8Eeeu0xXdbba9frFj0=OqFfea0dXdd9vqai=hGuQ8kuc9pgc9s8qqaq=dirpe0xb9q8qiLsFr0=vr0=vr0dc8meaabaqaciaacaGaaeqabaqabeGadaaakeaacqWGZbWCdaqhaaWcbaGaemyAaKMaemOAaOgabaGaeGOmaidaaaaa@31F2@ s is unnecessary for URMA [14], but whether the uncertainty of the sij2
 MathType@MTEF@5@5@+=feaafiart1ev1aaatCvAUfKttLearuWrP9MDH5MBPbIqV92AaeXatLxBI9gBaebbnrfifHhDYfgasaacH8akY=wiFfYdH8Gipec8Eeeu0xXdbba9frFj0=OqFfea0dXdd9vqai=hGuQ8kuc9pgc9s8qqaq=dirpe0xb9q8qiLsFr0=vr0=vr0dc8meaabaqaciaacaGaaeqabaqabeGadaaakeaacqWGZbWCdaqhaaWcbaGaemyAaKMaemOAaOgabaGaeGOmaidaaaaa@31F2@ s and *ρ*_*Wi *_s should be incorporated in BRMA is yet to be examined. This issue is outside the scope of this paper but we note that a Bayesian framework is particularly flexible for incorporating such uncertainty [15].

### Within- and between-study correlation

The *within-study correlation*, *ρ*_*Wi*_, indicates whether *Y*_*i*1 _and *Y*_*i*2 _are correlated within a study, and these *ρ*_*Wi *_s are usually assumed known. For the telomerase data the *ρ*_*Wi *_s might be assumed to be zero because sensitivity and specificity values are calculated independently in a study using different sets of patients. In other BRMA applications the *ρ*_*Wi *_s can be non-zero, for example where the two endpoints are overall and disease-free survival [10]. In practice it may be difficult to obtain the value of non-zero *ρ*_*Wi *_s, although it can be done as evident in Berkey et al. [1] and the 'motivating example 2' below [7]. Suggestions for limiting the problem of unavailable *ρ*_*Wi *_s have been proposed [15-17], and this issue is considered further in the discussion.

The *between-study correlation*, *ρ*_*B*_, is not generally known and has to be estimated when fitting the BRMA. It indicates how the underlying *true *values, i.e. the *θ*_*i*1_s and the *θ*_*i*2 _s, are related across studies. It may be caused by differences across studies in patient-level characteristics, such as age and stage of disease, or changes in study-level characteristics, such as the threshold level in diagnostic studies

### Motivating example 2 – the CD4 data

Daniels and Hughes [7] assess whether the change in CD4 cell count is a surrogate for time to either development of AIDS or death in drug trials of patients with HIV. They consider between-treatment-arm log-hazard ratios of time to onset of AIDS or death (*Y*_*i*1_), and between-treatment-arm differences in mean changes in CD4 count (*Y*_*i*2_) from pre-treatment baseline to about six months. Fifteen relevant trials were identified. Some of the trials involved three or four treatment arms, but to enable application to BRMA here we only consider outcome differences between the control arm and the first treatment arm in the reported dataset [7]. All fifteen trials provided complete data, including the within-study correlations which were quite small, varying between -0.22 and 0.17 with a mean of -0.08.

### Estimation

In our analyses of equation (1) in this paper the between-study parameters (i.e. τ12
 MathType@MTEF@5@5@+=feaafiart1ev1aaatCvAUfKttLearuWrP9MDH5MBPbIqV92AaeXatLxBI9gBaebbnrfifHhDYfgasaacH8akY=wiFfYdH8Gipec8Eeeu0xXdbba9frFj0=OqFfea0dXdd9vqai=hGuQ8kuc9pgc9s8qqaq=dirpe0xb9q8qiLsFr0=vr0=vr0dc8meaabaqaciaacaGaaeqabaqabeGadaaakeaaiiGacqWFepaDdaqhaaWcbaGaeGymaedabaGaeGOmaidaaaaa@3087@, τ22
 MathType@MTEF@5@5@+=feaafiart1ev1aaatCvAUfKttLearuWrP9MDH5MBPbIqV92AaeXatLxBI9gBaebbnrfifHhDYfgasaacH8akY=wiFfYdH8Gipec8Eeeu0xXdbba9frFj0=OqFfea0dXdd9vqai=hGuQ8kuc9pgc9s8qqaq=dirpe0xb9q8qiLsFr0=vr0=vr0dc8meaabaqaciaacaGaaeqabaqabeGadaaakeaaiiGacqWFepaDdaqhaaWcbaGaeGOmaidabaGaeGOmaidaaaaa@3089@ and *ρ*_*B*_) and the two pooled values (*β*_1 _and *β*_2_) are estimated iteratively using restricted maximum likelihood (REML) in SAS Proc Mixed, as described elsewhere [4]. Unless otherwise stated, we also use Cholesky decomposition [18] of **Ω **to ensure that this matrix is estimated to be positive semi-definite and therefore that the between-study correlation estimate, ρ^
 MathType@MTEF@5@5@+=feaafiart1ev1aaatCvAUfKttLearuWrP9MDH5MBPbIqV92AaeXatLxBI9gBaebbnrfifHhDYfgasaacH8akY=wiFfYdH8Gipec8Eeeu0xXdbba9frFj0=OqFfea0dXdd9vqai=hGuQ8kuc9pgc9s8qqaq=dirpe0xb9q8qiLsFr0=vr0=vr0dc8meaabaqaciaacaGaaeqabaqabeGadaaakeaaiiGacuWFbpGCgaqcaaaa@2E83@_*B*_, is in the range [-1,1]. Cholesky decomposition of **Ω **also helps ensure convergence when ρ^
 MathType@MTEF@5@5@+=feaafiart1ev1aaatCvAUfKttLearuWrP9MDH5MBPbIqV92AaeXatLxBI9gBaebbnrfifHhDYfgasaacH8akY=wiFfYdH8Gipec8Eeeu0xXdbba9frFj0=OqFfea0dXdd9vqai=hGuQ8kuc9pgc9s8qqaq=dirpe0xb9q8qiLsFr0=vr0=vr0dc8meaabaqaciaacaGaaeqabaqabeGadaaakeaaiiGacuWFbpGCgaqcaaaa@2E83@_*B *_is very close to 1 or -1.

### Analytic consideration of the between-study covariance parameters

Estimation and inference in classical linear mixed models are based on the marginal model, which for equation (1) is the bivariate normal model with variance-covariance **δ**_**i **_+ **Ω**. Assume for the sake of simplicity that the within-study covariance matrix **δ**_*i *_= **δ **for all studies. Then the covariance matrix of the observed *Y*_*i*1 _s and *Y*_*i*2 _s is given by **V **= **δ **+ **Ω**, where **δ **is known. Now, this puts (severe) restrictions on **V**, namely that **V **- **δ **is a covariance matrix, that is non-negative definite. If the estimated **V **does not satisfy this restriction, the maximum likelihood estimate of **Ω **will be truncated on the boundary of its parameter space. This means that if **Ω **is diagonal (as for URMA) the maximum likelihood estimate on the boundary will have one or both of the between-study variances equal to zero; else if **Ω **is non-diagonal (as for BRMA) then either one or both of the between-study variances equals zero or else the between-study correlation equals -1 or +1. In meta-analysis a between-study variance estimate of zero is well-understood, but a between-study correlation estimate of -1 or +1 is likely to be less familiar to practitioners. Thompson et al. [6] refer to this issue as 'poor estimation' but it perhaps should rather be considered a natural consequence of the sensible restrictions imposed on **V**, and one that prevents a variance estimate < 0 or a correlation estimate > +1 or < -1, as might otherwise be obtained.

### Rationale for the research in this paper

In this paper, to aid practitioners we will further assess the normal BRMA model and the role of between-study correlation. In particular, we aim to identify the situations when ρ^
 MathType@MTEF@5@5@+=feaafiart1ev1aaatCvAUfKttLearuWrP9MDH5MBPbIqV92AaeXatLxBI9gBaebbnrfifHhDYfgasaacH8akY=wiFfYdH8Gipec8Eeeu0xXdbba9frFj0=OqFfea0dXdd9vqai=hGuQ8kuc9pgc9s8qqaq=dirpe0xb9q8qiLsFr0=vr0=vr0dc8meaabaqaciaacaGaaeqabaqabeGadaaakeaaiiGacuWFbpGCgaqcaaaa@2E83@_*B *_is likely to be +1 or -1 and examine the impact this has on the pooled and between-study variance estimates. We will also evaluate the benefits of BRMA over two separate URMAs, the more common approach in practice, and explore extensions to a generalised BRMA model for meta-analysis of proportions. To achieve these goals we firstly apply the normal BRMA of equation (1) to the two motivating examples. We then perform a simulation study of the normal BRMA model, as described below. The generalised BRMA model is then introduced and assessed in relation to the normal BRMA model and two separate generalised URMAs (see Results).

### A simulation study to assess BRMA and the between-study correlation

We carried out a simulation study of the general normal model for BRMA in 11 scenarios, labelled (i) to (xi) (Table 2). Each of scenarios (i) to (xi) relates to a different but realistic specification of equation (1). Scenarios (i) to (vi) consider complete data, as in the telomerase example, whereas scenarios (vii) to (vi) consider when some data are missing at random across studies, as assumed in the BRMA of Thompson et al. [6]. The scenarios also vary in the relative sizes of the within- and between-study correlations, and also the within- and between-study variation. For example, scenarios (i) to (iv) involve within-study variances similar in size to the between-study variances, as observed in prognostic studies [10], and in scenario (vi) there is one relatively low and one relatively high between-study variance, as for the CD4 dataset. The sizes of the meta-analysis were either *n = 5 *or *n = 50 *studies for complete data, and either *n = 10 *or *n = 50 *for missing data. Our method of simulation was deliberately chosen to be similar to that previously used by Berkey et al. [1] and Sohn [19]. As an example, we now describe the simulation procedure for scenario (i) with *n = 50*.

**Table 2:** Scenarios used in the simulations based on equation (1)

	Pooled values		Between-study variances		Within- and between-study correlation		Within-study variation		
**Scenario**	*β*_1_	*β*_2_	τ12 MathType@MTEF@5@5@+=feaafiart1ev1aaatCvAUfKttLearuWrP9MDH5MBPbIqV92AaeXatLxBI9gBaebbnrfifHhDYfgasaacH8akY=wiFfYdH8Gipec8Eeeu0xXdbba9frFj0=OqFfea0dXdd9vqai=hGuQ8kuc9pgc9s8qqaq=dirpe0xb9q8qiLsFr0=vr0=vr0dc8meaabaqaciaacaGaaeqabaqabeGadaaakeaaiiGacqWFepaDdaqhaaWcbaGaeGymaedabaGaeGOmaidaaaaa@3087@	τ22 MathType@MTEF@5@5@+=feaafiart1ev1aaatCvAUfKttLearuWrP9MDH5MBPbIqV92AaeXatLxBI9gBaebbnrfifHhDYfgasaacH8akY=wiFfYdH8Gipec8Eeeu0xXdbba9frFj0=OqFfea0dXdd9vqai=hGuQ8kuc9pgc9s8qqaq=dirpe0xb9q8qiLsFr0=vr0=vr0dc8meaabaqaciaacaGaaeqabaqabeGadaaakeaaiiGacqWFepaDdaqhaaWcbaGaeGOmaidabaGaeGOmaidaaaaa@3089@	*ρ*_*Wi*_	*ρ*_*B*_	Median value of the sij2 MathType@MTEF@5@5@+=feaafiart1ev1aaatCvAUfKttLearuWrP9MDH5MBPbIqV92AaeXatLxBI9gBaebbnrfifHhDYfgasaacH8akY=wiFfYdH8Gipec8Eeeu0xXdbba9frFj0=OqFfea0dXdd9vqai=hGuQ8kuc9pgc9s8qqaq=dirpe0xb9q8qiLsFr0=vr0=vr0dc8meaabaqaciaacaGaaeqabaqabeGadaaakeaacqWGZbWCdaqhaaWcbaGaemyAaKMaemOAaOgabaGaeGOmaidaaaaa@31F2@ s:		Description

*Complete data*							*n = 50*	*n = 5*	
**(i)**	0	2	0.25	0.25	0	0	0.254	0.147	Zero correlation; within-study variation similar to between-study variation
**(ii)**	0	2	0.25	0.25	0	0.8	0.254	0.147	No within-study correlation but high between-study correlation; within-study variation similar to between-study variation
**(iii)**	0	2	0.25	0.25	0.8	0	0.254	0.147	High within-study correlation but no between-study correlation; within-study variation similar to between-study variation
**(iv)**	0	2	0.25	0.25	0.8	0.8	0.254	0.147	High within- and between-study correlation; within-study variation similar to between-study variation
**(v)**	0	2	0.0025	0.0025	0.8	0.8	0.254	0.147	High within- and between-study correlation; within-study variation large relative to between-study variation
**(vi)**	0	2	0.0025	1.5	0.8	0.8	0.254	0.147	High within- and between-study correlation; within-study variation large (for endpoint 1) and small (for endpoint 2) relative to between-study variation
**(vii)**	0	2	1.5	1.5	0.8	0.8	0.254	0.147	High within- and between-study correlation; within-study variation small relative to between-study variation

*Missing data*							*n = 50*	*n = 10*	
**(viii)**	0	2	1.5	1.5	0	0.8	0.244	0.183	No within-study correlation but high between-study correlation; within-study variance small relative to between-study variance
**(ix)**	0	2	0.25	0.25	0	0.8	0.244	0.183	No within-study correlation but high between-study correlation; within-study variance similar to between-study variance
**(x)**	0	2	1.5	1.5	0.8	0.8	0.244	0.183	High within- and between-study correlation; within-study variance small relative to between-study variance
**(xi)**	0	2	0.25	0.25	0.8	0.8	0.244	0.183	High within- and between-study correlation; within-study variance similar to between-study variance

### Description of the simulation procedure for scenario (i) with *n = 50*

#### Generation of a dataset of 1000 meta-analyses

We chose *β*_1 _= 0 in order to reflect little clinical benefit (e.g. a sensitivity of 50%) and in contrast *β*_2 _= 2 (e.g. a specificity of 88%). The within study variances required, i.e. the 50 si12
 MathType@MTEF@5@5@+=feaafiart1ev1aaatCvAUfKttLearuWrP9MDH5MBPbIqV92AaeXatLxBI9gBaebbnrfifHhDYfgasaacH8akY=wiFfYdH8Gipec8Eeeu0xXdbba9frFj0=OqFfea0dXdd9vqai=hGuQ8kuc9pgc9s8qqaq=dirpe0xb9q8qiLsFr0=vr0=vr0dc8meaabaqaciaacaGaaeqabaqabeGadaaakeaacqWGZbWCdaqhaaWcbaGaemyAaKMaeGymaedabaGaeGOmaidaaaaa@3185@ s and 50 si22
 MathType@MTEF@5@5@+=feaafiart1ev1aaatCvAUfKttLearuWrP9MDH5MBPbIqV92AaeXatLxBI9gBaebbnrfifHhDYfgasaacH8akY=wiFfYdH8Gipec8Eeeu0xXdbba9frFj0=OqFfea0dXdd9vqai=hGuQ8kuc9pgc9s8qqaq=dirpe0xb9q8qiLsFr0=vr0=vr0dc8meaabaqaciaacaGaaeqabaqabeGadaaakeaacqWGZbWCdaqhaaWcbaGaemyAaKMaeGOmaidabaGaeGOmaidaaaaa@3187@ s, were each found by sampling from a *N*(0.25,0.50) distribution and squaring the value obtained. This produced a median sij2
 MathType@MTEF@5@5@+=feaafiart1ev1aaatCvAUfKttLearuWrP9MDH5MBPbIqV92AaeXatLxBI9gBaebbnrfifHhDYfgasaacH8akY=wiFfYdH8Gipec8Eeeu0xXdbba9frFj0=OqFfea0dXdd9vqai=hGuQ8kuc9pgc9s8qqaq=dirpe0xb9q8qiLsFr0=vr0=vr0dc8meaabaqaciaacaGaaeqabaqabeGadaaakeaacqWGZbWCdaqhaaWcbaGaemyAaKMaemOAaOgabaGaeGOmaidaaaaa@31F2@ of 0.25 and an interquartile range of 0.7, values similar to those for the telomerase data (median sij2
 MathType@MTEF@5@5@+=feaafiart1ev1aaatCvAUfKttLearuWrP9MDH5MBPbIqV92AaeXatLxBI9gBaebbnrfifHhDYfgasaacH8akY=wiFfYdH8Gipec8Eeeu0xXdbba9frFj0=OqFfea0dXdd9vqai=hGuQ8kuc9pgc9s8qqaq=dirpe0xb9q8qiLsFr0=vr0=vr0dc8meaabaqaciaacaGaaeqabaqabeGadaaakeaacqWGZbWCdaqhaaWcbaGaemyAaKMaemOAaOgabaGaeGOmaidaaaaa@31F2@ = 0.21) and for the BRMA of Thompson et al. [6] (median sij2
 MathType@MTEF@5@5@+=feaafiart1ev1aaatCvAUfKttLearuWrP9MDH5MBPbIqV92AaeXatLxBI9gBaebbnrfifHhDYfgasaacH8akY=wiFfYdH8Gipec8Eeeu0xXdbba9frFj0=OqFfea0dXdd9vqai=hGuQ8kuc9pgc9s8qqaq=dirpe0xb9q8qiLsFr0=vr0=vr0dc8meaabaqaciaacaGaaeqabaqabeGadaaakeaacqWGZbWCdaqhaaWcbaGaemyAaKMaemOAaOgabaGaeGOmaidaaaaa@31F2@ = 0.28, interquartile range = 0.76). The between-study variances, τ12
 MathType@MTEF@5@5@+=feaafiart1ev1aaatCvAUfKttLearuWrP9MDH5MBPbIqV92AaeXatLxBI9gBaebbnrfifHhDYfgasaacH8akY=wiFfYdH8Gipec8Eeeu0xXdbba9frFj0=OqFfea0dXdd9vqai=hGuQ8kuc9pgc9s8qqaq=dirpe0xb9q8qiLsFr0=vr0=vr0dc8meaabaqaciaacaGaaeqabaqabeGadaaakeaaiiGacqWFepaDdaqhaaWcbaGaeGymaedabaGaeGOmaidaaaaa@3087@ and τ22
 MathType@MTEF@5@5@+=feaafiart1ev1aaatCvAUfKttLearuWrP9MDH5MBPbIqV92AaeXatLxBI9gBaebbnrfifHhDYfgasaacH8akY=wiFfYdH8Gipec8Eeeu0xXdbba9frFj0=OqFfea0dXdd9vqai=hGuQ8kuc9pgc9s8qqaq=dirpe0xb9q8qiLsFr0=vr0=vr0dc8meaabaqaciaacaGaaeqabaqabeGadaaakeaaiiGacqWFepaDdaqhaaWcbaGaeGOmaidabaGaeGOmaidaaaaa@3089@, were chosen to be 0.25 in scenario (i), which meant that they were similar in size to the median within-study variances. The within and between-study correlations were both set to zero for this scenario. All these choices were substituted into equation (1) and 1000 meta-analyses each of 50 studies were generated. Calculations were performed in S-Plus using the 'rmvnorm' function for generating bivariate normal values (code available upon request).

#### Estimation using the dataset of 1000 meta-analyses

Each of the 1000 meta-analyses in scenario (i) were analysed separately by:

• fitting two separate URMAs as in equation (2) (where *ρ*_*B *_= 0) using REML to estimate *β*_1_, *β*_2_, τ12
 MathType@MTEF@5@5@+=feaafiart1ev1aaatCvAUfKttLearuWrP9MDH5MBPbIqV92AaeXatLxBI9gBaebbnrfifHhDYfgasaacH8akY=wiFfYdH8Gipec8Eeeu0xXdbba9frFj0=OqFfea0dXdd9vqai=hGuQ8kuc9pgc9s8qqaq=dirpe0xb9q8qiLsFr0=vr0=vr0dc8meaabaqaciaacaGaaeqabaqabeGadaaakeaaiiGacqWFepaDdaqhaaWcbaGaeGymaedabaGaeGOmaidaaaaa@3087@ and τ22
 MathType@MTEF@5@5@+=feaafiart1ev1aaatCvAUfKttLearuWrP9MDH5MBPbIqV92AaeXatLxBI9gBaebbnrfifHhDYfgasaacH8akY=wiFfYdH8Gipec8Eeeu0xXdbba9frFj0=OqFfea0dXdd9vqai=hGuQ8kuc9pgc9s8qqaq=dirpe0xb9q8qiLsFr0=vr0=vr0dc8meaabaqaciaacaGaaeqabaqabeGadaaakeaaiiGacqWFepaDdaqhaaWcbaGaeGOmaidabaGaeGOmaidaaaaa@3089@

• fitting a BRMA as in equation (1) using REML to estimate *β*_1_, *β*_2_, τ12
 MathType@MTEF@5@5@+=feaafiart1ev1aaatCvAUfKttLearuWrP9MDH5MBPbIqV92AaeXatLxBI9gBaebbnrfifHhDYfgasaacH8akY=wiFfYdH8Gipec8Eeeu0xXdbba9frFj0=OqFfea0dXdd9vqai=hGuQ8kuc9pgc9s8qqaq=dirpe0xb9q8qiLsFr0=vr0=vr0dc8meaabaqaciaacaGaaeqabaqabeGadaaakeaaiiGacqWFepaDdaqhaaWcbaGaeGymaedabaGaeGOmaidaaaaa@3087@, τ22
 MathType@MTEF@5@5@+=feaafiart1ev1aaatCvAUfKttLearuWrP9MDH5MBPbIqV92AaeXatLxBI9gBaebbnrfifHhDYfgasaacH8akY=wiFfYdH8Gipec8Eeeu0xXdbba9frFj0=OqFfea0dXdd9vqai=hGuQ8kuc9pgc9s8qqaq=dirpe0xb9q8qiLsFr0=vr0=vr0dc8meaabaqaciaacaGaaeqabaqabeGadaaakeaaiiGacqWFepaDdaqhaaWcbaGaeGOmaidabaGaeGOmaidaaaaa@3089@, and *ρ*_*B*_

The 1000 BRMA estimates and the corresponding 1000 URMA estimates from scenario (i) were then compared by calculating:

• average parameter estimates across all the simulations (to check for bias)

• coverage of the 95% confidence intervals for *β*_1 _and *β*_2_

• average standard error and mean-square error (MSE) of *β*_1 _and *β*_2_

• the number of occasions ρ^
 MathType@MTEF@5@5@+=feaafiart1ev1aaatCvAUfKttLearuWrP9MDH5MBPbIqV92AaeXatLxBI9gBaebbnrfifHhDYfgasaacH8akY=wiFfYdH8Gipec8Eeeu0xXdbba9frFj0=OqFfea0dXdd9vqai=hGuQ8kuc9pgc9s8qqaq=dirpe0xb9q8qiLsFr0=vr0=vr0dc8meaabaqaciaacaGaaeqabaqabeGadaaakeaaiiGacuWFbpGCgaqcaaaa@2E83@_*B *_was equal to +1 or -1 in the BRMA

To assess coverage, the 95% confidence intervals for *β*_*j *_were calculated using:

β^j±(tnj−1(0.05)∗var(β^j)),
 MathType@MTEF@5@5@+=feaafiart1ev1aaatCvAUfKttLearuWrP9MDH5MBPbIqV92AaeXatLxBI9gBaebbnrfifHhDYfgasaacH8akY=wiFfYdH8Gipec8Eeeu0xXdbba9frFj0=OqFfea0dXdd9vqai=hGuQ8kuc9pgc9s8qqaq=dirpe0xb9q8qiLsFr0=vr0=vr0dc8meaabaqaciaacaGaaeqabaqabeGadaaakeaaiiGacuWFYoGygaqcamaaBaaaleaacqWGQbGAaeqaaOGaeyySae7aaeWaaeaacqWG0baDdaWgaaWcbaGaemOBa42aaSbaaWqaaiabdQgaQbqabaWccqGHsislcqaIXaqmaeqaaOGaeiikaGIaeGimaaJaeiOla4IaeGimaaJaeGynauJaeiykaKIaey4fIOYaaOaaaeaaieGacqGF2bGDcqGFHbqycqGFYbGCcqGGOaakcuWFYoGygaqcamaaBaaaleaacqWGQbGAaeqaaOGaeiykaKcaleqaaaGccaGLOaGaayzkaaGaeiilaWcaaa@4A64@

with *n*_*j *_the number of studies providing endpoint *j*. This *t*-distribution is commonly used in the meta-analysis literature, although it is only an approximation [20].

### Description of the simulation procedure for scenarios (ii) to (xi)

Simulations in the other scenarios followed in the same manner as described above but with the data generated from the parameter values specific to each scenario as given in Table 2. For those missing data simulations of scenarios (viii) to (xi) we simulated data as described for complete data, except that for each generated meta-analysis we removed the data for the second endpoint in a randomly selected 50% of studies. So, for example, with *n = 50 *in scenario (viii) each of the 1000 simulated meta-analyses contained 25 studies with complete data and 25 studies with data for the first endpoint only.

## Results

### Application to the telomerase and CD4 data

**Figure 1:**
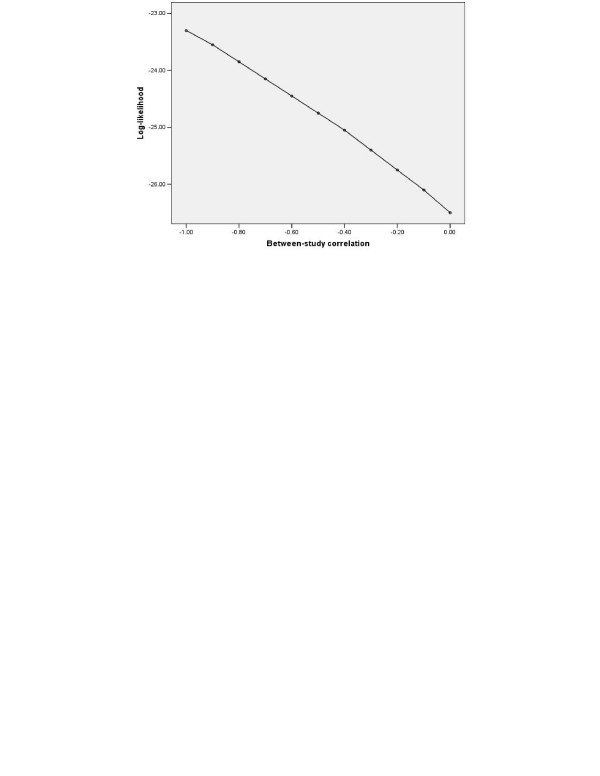
Profile log-likelihood for the between-study correlation from the general normal BRMA of the telomerase data.

The normal model for BRMA (equation (1)) and then two separate URMAs (equation (2)) were applied to the telomerase data. Both approaches gave a pooled sensitivity of about 76% and a pooled specificity of about 88% (Table 3). The BRMA gave a between-study correlation estimate of -1 but the profile likelihood reveals that there is little information regarding *ρ*_*B *_with the log-likelihood gradually increasing as ρ^
 MathType@MTEF@5@5@+=feaafiart1ev1aaatCvAUfKttLearuWrP9MDH5MBPbIqV92AaeXatLxBI9gBaebbnrfifHhDYfgasaacH8akY=wiFfYdH8Gipec8Eeeu0xXdbba9frFj0=OqFfea0dXdd9vqai=hGuQ8kuc9pgc9s8qqaq=dirpe0xb9q8qiLsFr0=vr0=vr0dc8meaabaqaciaacaGaaeqabaqabeGadaaakeaaiiGacuWFbpGCgaqcaaaa@2E83@_*B *_approaches -1 (Figure 1), the end of its parameter space. Interestingly, the between-study variances were estimated to be somewhat larger in the BRMA than the URMA, and the standard errors of the pooled estimates were also slightly larger in the BRMA; just the opposite of what one might expect from a bivariate analysis utilising large correlation [10]. A similar finding was observed upon application of BRMA and URMA to the CD4 data. The BRMA again gave a between-study correlation estimate of -1 and both between-study variances were estimated somewhat larger in the BRMA than the URMA, as were the standard errors of the pooled estimates.

**Table 3:** URMA and BRMA results for the telomerase and CD4 datasets

**Dataset**	**Model**	Pooled value endpoint 1 β^ MathType@MTEF@5@5@+=feaafiart1ev1aaatCvAUfKttLearuWrP9MDH5MBPbIqV92AaeXatLxBI9gBaebbnrfifHhDYfgasaacH8akY=wiFfYdH8Gipec8Eeeu0xXdbba9frFj0=OqFfea0dXdd9vqai=hGuQ8kuc9pgc9s8qqaq=dirpe0xb9q8qiLsFr0=vr0=vr0dc8meaabaqaciaacaGaaeqabaqabeGadaaakeaaiiGacuWFYoGygaqcaaaa@2E64@_1 _(s.e.)	Between-study variance endpoint 2 τ^12 MathType@MTEF@5@5@+=feaafiart1ev1aaatCvAUfKttLearuWrP9MDH5MBPbIqV92AaeXatLxBI9gBaebbnrfifHhDYfgasaacH8akY=wiFfYdH8Gipec8Eeeu0xXdbba9frFj0=OqFfea0dXdd9vqai=hGuQ8kuc9pgc9s8qqaq=dirpe0xb9q8qiLsFr0=vr0=vr0dc8meaabaqaciaacaGaaeqabaqabeGadaaakeaaiiGacuWFepaDgaqcamaaDaaaleaacqaIXaqmaeaacqaIYaGmaaaaaa@3097@	Pooled value endpoint 2 β^ MathType@MTEF@5@5@+=feaafiart1ev1aaatCvAUfKttLearuWrP9MDH5MBPbIqV92AaeXatLxBI9gBaebbnrfifHhDYfgasaacH8akY=wiFfYdH8Gipec8Eeeu0xXdbba9frFj0=OqFfea0dXdd9vqai=hGuQ8kuc9pgc9s8qqaq=dirpe0xb9q8qiLsFr0=vr0=vr0dc8meaabaqaciaacaGaaeqabaqabeGadaaakeaaiiGacuWFYoGygaqcaaaa@2E64@_2 _(s.e.)	Between-study variance endpoint 2 τ^22 MathType@MTEF@5@5@+=feaafiart1ev1aaatCvAUfKttLearuWrP9MDH5MBPbIqV92AaeXatLxBI9gBaebbnrfifHhDYfgasaacH8akY=wiFfYdH8Gipec8Eeeu0xXdbba9frFj0=OqFfea0dXdd9vqai=hGuQ8kuc9pgc9s8qqaq=dirpe0xb9q8qiLsFr0=vr0=vr0dc8meaabaqaciaacaGaaeqabaqabeGadaaakeaaiiGacuWFepaDgaqcamaaDaaaleaacqaIYaGmaeaacqaIYaGmaaaaaa@3099@	Between-study correlation ρ^ MathType@MTEF@5@5@+=feaafiart1ev1aaatCvAUfKttLearuWrP9MDH5MBPbIqV92AaeXatLxBI9gBaebbnrfifHhDYfgasaacH8akY=wiFfYdH8Gipec8Eeeu0xXdbba9frFj0=OqFfea0dXdd9vqai=hGuQ8kuc9pgc9s8qqaq=dirpe0xb9q8qiLsFr0=vr0=vr0dc8meaabaqaciaacaGaaeqabaqabeGadaaakeaaiiGacuWFbpGCgaqcaaaa@2E83@_*B*_
**Telomerase**	Normal URMA	1.155 (0.186)	0.186	1.964 (0.541)	2.386	NA
	Normal BRMA	1.166 (0.186)	0.202	2.058 (0.554)	2.584	-1.0
	Generalised URMA	1.182 (0.176)	0.155	2.215 (0.578)	2.680	NA

**CD4**	Normal URMA	-0.049 (0.0695)	0.025	17.300 (5.561)	379.73	NA
	Normal BRMA	-0.109 (0.0748)	0.048	18.314 (5.740)	412.96	-1.0

s.e. = standard error; NA = not applicable; 95% confidence interval calculated using t-distribution with 9 degrees of freedom. Restricted maximum likelihood estimation was used for the normal models, whereas maximum likelihood estimation was used for the generalised model.

The question is thus posed: is the estimation of *ρ*_*B *_at the boundary of its parameter space adversely influencing the other BRMA parameter estimates and, if so, how (e.g. does it introduce bias)? Also, in terms of the individual pooled estimates, the telomerase and CD4 examples indicate little benefit of BRMA over two separate URMAs, despite the utilisation of correlation; but is this generally true and in what situations should a BRMA be preferred? To understand the answers to these important questions, it is helpful to now consider the results from our simulation study.

We note at this point that, for both telomerase and CD4, we also tried estimation of BRMA using an unstructured form of **Ω**, rather than using Cholesky decomposition of **Ω **as previously. Interestingly, this approach produced non-sensical between-study correlation estimates of -1.12 and -1.074 for the telomerase and CD4 datasets respectively. This emphasises the importance of a boundary constraint on *ρ*_*B *_as imposed by the Cholesky decomposition.

### Simulation results

Table 4 includes the simulation results for scenarios (i), (ii), (viii) and (ix). The results for all other scenarios are provided in Appendix 1 (see Additional file 1). We now discuss the key findings.

**Table 4:** Simulation results of the normal BRMA and URMA models for scenarios (i), (ii), (viii), and (ix)

**Meta-analysis model**	***n***	**No. of the 1000 simulations that converged**	**Bias of mean **β^ MathType@MTEF@5@5@+=feaafiart1ev1aaatCvAUfKttLearuWrP9MDH5MBPbIqV92AaeXatLxBI9gBaebbnrfifHhDYfgasaacH8akY=wiFfYdH8Gipec8Eeeu0xXdbba9frFj0=OqFfea0dXdd9vqai=hGuQ8kuc9pgc9s8qqaq=dirpe0xb9q8qiLsFr0=vr0=vr0dc8meaabaqaciaacaGaaeqabaqabeGadaaakeaaiiGacuWFYoGygaqcaaaa@2E64@_1_	**Mean s.e. of **β^ MathType@MTEF@5@5@+=feaafiart1ev1aaatCvAUfKttLearuWrP9MDH5MBPbIqV92AaeXatLxBI9gBaebbnrfifHhDYfgasaacH8akY=wiFfYdH8Gipec8Eeeu0xXdbba9frFj0=OqFfea0dXdd9vqai=hGuQ8kuc9pgc9s8qqaq=dirpe0xb9q8qiLsFr0=vr0=vr0dc8meaabaqaciaacaGaaeqabaqabeGadaaakeaaiiGacuWFYoGygaqcaaaa@2E64@_1_	**MSE of **β^ MathType@MTEF@5@5@+=feaafiart1ev1aaatCvAUfKttLearuWrP9MDH5MBPbIqV92AaeXatLxBI9gBaebbnrfifHhDYfgasaacH8akY=wiFfYdH8Gipec8Eeeu0xXdbba9frFj0=OqFfea0dXdd9vqai=hGuQ8kuc9pgc9s8qqaq=dirpe0xb9q8qiLsFr0=vr0=vr0dc8meaabaqaciaacaGaaeqabaqabeGadaaakeaaiiGacuWFYoGygaqcaaaa@2E64@_1_	**Coverage of the 95% CIs for **β^ MathType@MTEF@5@5@+=feaafiart1ev1aaatCvAUfKttLearuWrP9MDH5MBPbIqV92AaeXatLxBI9gBaebbnrfifHhDYfgasaacH8akY=wiFfYdH8Gipec8Eeeu0xXdbba9frFj0=OqFfea0dXdd9vqai=hGuQ8kuc9pgc9s8qqaq=dirpe0xb9q8qiLsFr0=vr0=vr0dc8meaabaqaciaacaGaaeqabaqabeGadaaakeaaiiGacuWFYoGygaqcaaaa@2E64@_1_	**Bias of mean **β^ MathType@MTEF@5@5@+=feaafiart1ev1aaatCvAUfKttLearuWrP9MDH5MBPbIqV92AaeXatLxBI9gBaebbnrfifHhDYfgasaacH8akY=wiFfYdH8Gipec8Eeeu0xXdbba9frFj0=OqFfea0dXdd9vqai=hGuQ8kuc9pgc9s8qqaq=dirpe0xb9q8qiLsFr0=vr0=vr0dc8meaabaqaciaacaGaaeqabaqabeGadaaakeaaiiGacuWFYoGygaqcaaaa@2E64@_2_	**Mean s.e. of **β^ MathType@MTEF@5@5@+=feaafiart1ev1aaatCvAUfKttLearuWrP9MDH5MBPbIqV92AaeXatLxBI9gBaebbnrfifHhDYfgasaacH8akY=wiFfYdH8Gipec8Eeeu0xXdbba9frFj0=OqFfea0dXdd9vqai=hGuQ8kuc9pgc9s8qqaq=dirpe0xb9q8qiLsFr0=vr0=vr0dc8meaabaqaciaacaGaaeqabaqabeGadaaakeaaiiGacuWFYoGygaqcaaaa@2E64@_2_	**MSE of **β^ MathType@MTEF@5@5@+=feaafiart1ev1aaatCvAUfKttLearuWrP9MDH5MBPbIqV92AaeXatLxBI9gBaebbnrfifHhDYfgasaacH8akY=wiFfYdH8Gipec8Eeeu0xXdbba9frFj0=OqFfea0dXdd9vqai=hGuQ8kuc9pgc9s8qqaq=dirpe0xb9q8qiLsFr0=vr0=vr0dc8meaabaqaciaacaGaaeqabaqabeGadaaakeaaiiGacuWFYoGygaqcaaaa@2E64@_2_	**Coverage of the 95% CIs for **β^ MathType@MTEF@5@5@+=feaafiart1ev1aaatCvAUfKttLearuWrP9MDH5MBPbIqV92AaeXatLxBI9gBaebbnrfifHhDYfgasaacH8akY=wiFfYdH8Gipec8Eeeu0xXdbba9frFj0=OqFfea0dXdd9vqai=hGuQ8kuc9pgc9s8qqaq=dirpe0xb9q8qiLsFr0=vr0=vr0dc8meaabaqaciaacaGaaeqabaqabeGadaaakeaaiiGacuWFYoGygaqcaaaa@2E64@_2_	**Bias of mean **τ^12 MathType@MTEF@5@5@+=feaafiart1ev1aaatCvAUfKttLearuWrP9MDH5MBPbIqV92AaeXatLxBI9gBaebbnrfifHhDYfgasaacH8akY=wiFfYdH8Gipec8Eeeu0xXdbba9frFj0=OqFfea0dXdd9vqai=hGuQ8kuc9pgc9s8qqaq=dirpe0xb9q8qiLsFr0=vr0=vr0dc8meaabaqaciaacaGaaeqabaqabeGadaaakeaaiiGacuWFepaDgaqcamaaDaaaleaacqaIXaqmaeaacqaIYaGmaaaaaa@3097@**(no. of **τ^12 MathType@MTEF@5@5@+=feaafiart1ev1aaatCvAUfKttLearuWrP9MDH5MBPbIqV92AaeXatLxBI9gBaebbnrfifHhDYfgasaacH8akY=wiFfYdH8Gipec8Eeeu0xXdbba9frFj0=OqFfea0dXdd9vqai=hGuQ8kuc9pgc9s8qqaq=dirpe0xb9q8qiLsFr0=vr0=vr0dc8meaabaqaciaacaGaaeqabaqabeGadaaakeaaiiGacuWFepaDgaqcamaaDaaaleaacqaIXaqmaeaacqaIYaGmaaaaaa@3097@**= 0)**	**Bias of mean **τ^22 MathType@MTEF@5@5@+=feaafiart1ev1aaatCvAUfKttLearuWrP9MDH5MBPbIqV92AaeXatLxBI9gBaebbnrfifHhDYfgasaacH8akY=wiFfYdH8Gipec8Eeeu0xXdbba9frFj0=OqFfea0dXdd9vqai=hGuQ8kuc9pgc9s8qqaq=dirpe0xb9q8qiLsFr0=vr0=vr0dc8meaabaqaciaacaGaaeqabaqabeGadaaakeaaiiGacuWFepaDgaqcamaaDaaaleaacqaIYaGmaeaacqaIYaGmaaaaaa@3099@**(no. of **τ^22 MathType@MTEF@5@5@+=feaafiart1ev1aaatCvAUfKttLearuWrP9MDH5MBPbIqV92AaeXatLxBI9gBaebbnrfifHhDYfgasaacH8akY=wiFfYdH8Gipec8Eeeu0xXdbba9frFj0=OqFfea0dXdd9vqai=hGuQ8kuc9pgc9s8qqaq=dirpe0xb9q8qiLsFr0=vr0=vr0dc8meaabaqaciaacaGaaeqabaqabeGadaaakeaaiiGacuWFepaDgaqcamaaDaaaleaacqaIYaGmaeaacqaIYaGmaaaaaa@3099@**= 0)**	**Bias of mean **ρ^ MathType@MTEF@5@5@+=feaafiart1ev1aaatCvAUfKttLearuWrP9MDH5MBPbIqV92AaeXatLxBI9gBaebbnrfifHhDYfgasaacH8akY=wiFfYdH8Gipec8Eeeu0xXdbba9frFj0=OqFfea0dXdd9vqai=hGuQ8kuc9pgc9s8qqaq=dirpe0xb9q8qiLsFr0=vr0=vr0dc8meaabaqaciaacaGaaeqabaqabeGadaaakeaaiiGacuWFbpGCgaqcaaaa@2E83@_*B*_	**% of **ρ^ MathType@MTEF@5@5@+=feaafiart1ev1aaatCvAUfKttLearuWrP9MDH5MBPbIqV92AaeXatLxBI9gBaebbnrfifHhDYfgasaacH8akY=wiFfYdH8Gipec8Eeeu0xXdbba9frFj0=OqFfea0dXdd9vqai=hGuQ8kuc9pgc9s8qqaq=dirpe0xb9q8qiLsFr0=vr0=vr0dc8meaabaqaciaacaGaaeqabaqabeGadaaakeaaiiGacuWFbpGCgaqcaaaa@2E83@_*B *_**= -1**	**% of **ρ^ MathType@MTEF@5@5@+=feaafiart1ev1aaatCvAUfKttLearuWrP9MDH5MBPbIqV92AaeXatLxBI9gBaebbnrfifHhDYfgasaacH8akY=wiFfYdH8Gipec8Eeeu0xXdbba9frFj0=OqFfea0dXdd9vqai=hGuQ8kuc9pgc9s8qqaq=dirpe0xb9q8qiLsFr0=vr0=vr0dc8meaabaqaciaacaGaaeqabaqabeGadaaakeaaiiGacuWFbpGCgaqcaaaa@2E83@_*B *_**= 1**
**Scenario (i): Complete data – zero correlation; within-study variance similar to between-study variance**															
URMA	50	1000	-0.005	0.102	0.010	94.8%	0.001	0.107	0.0108	95.4%	-0.003 (0)	0.005 (1)	-	-	-
BRMA	50	1000	-0.005	0.101	0.010	94.7%	0.001	0.106	0.0108	95.5%	-0.003 (0)	0.005 (0)	-0.001	0.2%	0.4%
URMA	5	1000	-0.002	0.267	0.081	96.0%	-0.006	0.267	0.0887	94.0%	-0.006 (89)	0.015 (81)	-	-	-
BRMA	5	998	-0.002	0.274	0.081	96.7%	-0.006	0.269	0.0894	95.3%	0.008 (10)	0.024 (0)	-0.027	29.6%	29.0%
**Scenario (ii): Complete data – no within-study correlation, high between-study correlation; within-study variance similar to between-study variance**															
URMA	50	1000	-0.004	0.102	0.010	95.6%	0.001	0.106	0.0114	94.4%	0 (0)	-0.004 (1)	-	-	-
BRMA	50	1000	-0.004	0.100	0.010	95.3%	0	0.104	0.0107	95.4%	0.001 (0)	-0.001 (0)	-0.005	0%	25.2%
URMA	5	999	-0.002	0.271	0.077	97.3%	-0.005	0.263	0.0826	94.2%	0.004 (80)	0.005 (104)	-	-	-
BRMA	5	1000	-0.002	0.279	0.077	98.1%	-0.008	0.268	0.0819	95.7%	0.024 (15)	0.024 (0)	-0.161	10.3%	60.5%
**Scenario (viii): Missing data – no within-study correlation, high between-study correlation; within-study variance smaller than between-study variance**															
URMA	50	1000	-0.004	0.071	0.005	94.9%	0	0.099	0.0101	95.0%	-0.006 (0)	-0.005 (0)	-	-	-
BRMA	50	1000	-0.004	0.071	0.005	94.9%	0	0.082	0.0068	95.2%	-0.006 (0)	-0.007 (0)	-0.001	0%	0%
URMA	10	1000	-0.002	0.154	0.028	94.1%	-0.003	0.209	0.0576	93.7%	-0.006 (0)	-0.006 (0)	-	-	-
BRMA	10	1000	-0.002	0.154	0.028	94.1%	-0.001	0.174	0.0427	93.3%	-0.006 (0)	0.006 (0)	-0.040	0%	3.9%
**Scenario (ix): Missing data – no within-study correlation, high between-study correlation; within-study variance similar to between-study variance**															
URMA	50	1000	-0.004	0.102	0.010	95.6%	-0.001	0.145	0.0228	94.2%	0 (0)	-0.003 (6)	-	-	-
BRMA	50	1000	-0.004	0.101	0.010	95.8%	-0.003	0.137	0.0203	94.7%	0.001 (0)	0.003 (0)	-0.012	0.1%	35.6%
URMA	10	1000	-0.001	0.218	0.045	93.9%	-0.005	0.263	0.0825	94.2%	0.006 (45)	0.005 (84)	-	-	-
BRMA	10	997	-0.001	0.222	0.045	96.5%	-0.007	0.255	0.0797	95.6%	0.020 (0)	0.025 (0)	-0.164	10.1%	60.2%

MSE = mean-square-error, *n *= number of studies in each meta-analysis, CIs = confidence intervals, s.e. = standard error;

#### Between-study correlation

One can see from Table 4 that the normal model for BRMA often estimates the between-study correlation, *ρ*_*B*_, as either +1 or -1, especially when the number of studies in the meta-analysis is small. For example, with *n = 5 *in scenario (ii), where the true *ρ*_*B *_was 0.8, 605 of the 1000 simulations (60.5%) gave ρ^
 MathType@MTEF@5@5@+=feaafiart1ev1aaatCvAUfKttLearuWrP9MDH5MBPbIqV92AaeXatLxBI9gBaebbnrfifHhDYfgasaacH8akY=wiFfYdH8Gipec8Eeeu0xXdbba9frFj0=OqFfea0dXdd9vqai=hGuQ8kuc9pgc9s8qqaq=dirpe0xb9q8qiLsFr0=vr0=vr0dc8meaabaqaciaacaGaaeqabaqabeGadaaakeaaiiGacuWFbpGCgaqcaaaa@2E83@_*B *_equal to +1 and 103 simulations (10.3%) gave ρ^
 MathType@MTEF@5@5@+=feaafiart1ev1aaatCvAUfKttLearuWrP9MDH5MBPbIqV92AaeXatLxBI9gBaebbnrfifHhDYfgasaacH8akY=wiFfYdH8Gipec8Eeeu0xXdbba9frFj0=OqFfea0dXdd9vqai=hGuQ8kuc9pgc9s8qqaq=dirpe0xb9q8qiLsFr0=vr0=vr0dc8meaabaqaciaacaGaaeqabaqabeGadaaakeaaiiGacuWFbpGCgaqcaaaa@2E83@_*B *_equal to -1. This led to a mean value of ρ^
 MathType@MTEF@5@5@+=feaafiart1ev1aaatCvAUfKttLearuWrP9MDH5MBPbIqV92AaeXatLxBI9gBaebbnrfifHhDYfgasaacH8akY=wiFfYdH8Gipec8Eeeu0xXdbba9frFj0=OqFfea0dXdd9vqai=hGuQ8kuc9pgc9s8qqaq=dirpe0xb9q8qiLsFr0=vr0=vr0dc8meaabaqaciaacaGaaeqabaqabeGadaaakeaaiiGacuWFbpGCgaqcaaaa@2E83@_*B *_equal to 0.639, a downward bias of about 20%. However, this downward bias is not in itself a concern because it is simply caused by the maximum likelihood estimator sensibly truncating ρ^
 MathType@MTEF@5@5@+=feaafiart1ev1aaatCvAUfKttLearuWrP9MDH5MBPbIqV92AaeXatLxBI9gBaebbnrfifHhDYfgasaacH8akY=wiFfYdH8Gipec8Eeeu0xXdbba9frFj0=OqFfea0dXdd9vqai=hGuQ8kuc9pgc9s8qqaq=dirpe0xb9q8qiLsFr0=vr0=vr0dc8meaabaqaciaacaGaaeqabaqabeGadaaakeaaiiGacuWFbpGCgaqcaaaa@2E83@_*B *_at +1 and -1, which improves the mean-square error of ρ^
 MathType@MTEF@5@5@+=feaafiart1ev1aaatCvAUfKttLearuWrP9MDH5MBPbIqV92AaeXatLxBI9gBaebbnrfifHhDYfgasaacH8akY=wiFfYdH8Gipec8Eeeu0xXdbba9frFj0=OqFfea0dXdd9vqai=hGuQ8kuc9pgc9s8qqaq=dirpe0xb9q8qiLsFr0=vr0=vr0dc8meaabaqaciaacaGaaeqabaqabeGadaaakeaaiiGacuWFbpGCgaqcaaaa@2E83@_*B*_. Furthermore, ρ^
 MathType@MTEF@5@5@+=feaafiart1ev1aaatCvAUfKttLearuWrP9MDH5MBPbIqV92AaeXatLxBI9gBaebbnrfifHhDYfgasaacH8akY=wiFfYdH8Gipec8Eeeu0xXdbba9frFj0=OqFfea0dXdd9vqai=hGuQ8kuc9pgc9s8qqaq=dirpe0xb9q8qiLsFr0=vr0=vr0dc8meaabaqaciaacaGaaeqabaqabeGadaaakeaaiiGacuWFbpGCgaqcaaaa@2E83@_*B *_is clearly asymptotically unbiased, with the occurrence of ρ^
 MathType@MTEF@5@5@+=feaafiart1ev1aaatCvAUfKttLearuWrP9MDH5MBPbIqV92AaeXatLxBI9gBaebbnrfifHhDYfgasaacH8akY=wiFfYdH8Gipec8Eeeu0xXdbba9frFj0=OqFfea0dXdd9vqai=hGuQ8kuc9pgc9s8qqaq=dirpe0xb9q8qiLsFr0=vr0=vr0dc8meaabaqaciaacaGaaeqabaqabeGadaaakeaaiiGacuWFbpGCgaqcaaaa@2E83@_*B *_equal to -1 or +1 and thus the bias in mean ρ^
 MathType@MTEF@5@5@+=feaafiart1ev1aaatCvAUfKttLearuWrP9MDH5MBPbIqV92AaeXatLxBI9gBaebbnrfifHhDYfgasaacH8akY=wiFfYdH8Gipec8Eeeu0xXdbba9frFj0=OqFfea0dXdd9vqai=hGuQ8kuc9pgc9s8qqaq=dirpe0xb9q8qiLsFr0=vr0=vr0dc8meaabaqaciaacaGaaeqabaqabeGadaaakeaaiiGacuWFbpGCgaqcaaaa@2E83@_*B *_becoming increasingly less as the number of studies in the meta-analysis increases (Table 4). Interestingly though, the number of studies required to reduce the occurrence of ρ^
 MathType@MTEF@5@5@+=feaafiart1ev1aaatCvAUfKttLearuWrP9MDH5MBPbIqV92AaeXatLxBI9gBaebbnrfifHhDYfgasaacH8akY=wiFfYdH8Gipec8Eeeu0xXdbba9frFj0=OqFfea0dXdd9vqai=hGuQ8kuc9pgc9s8qqaq=dirpe0xb9q8qiLsFr0=vr0=vr0dc8meaabaqaciaacaGaaeqabaqabeGadaaakeaaiiGacuWFbpGCgaqcaaaa@2E83@_*B *_equal to -1 or +1 was far greater when the within-study variation was large relative to the between-study variation. For example, even with a large *n = 50 *studies in scenario (v), where the within-study variation was relatively large, 58% of the simulations gave a between-study correlation of -1 or +1.

These findings indicate why ρ^
 MathType@MTEF@5@5@+=feaafiart1ev1aaatCvAUfKttLearuWrP9MDH5MBPbIqV92AaeXatLxBI9gBaebbnrfifHhDYfgasaacH8akY=wiFfYdH8Gipec8Eeeu0xXdbba9frFj0=OqFfea0dXdd9vqai=hGuQ8kuc9pgc9s8qqaq=dirpe0xb9q8qiLsFr0=vr0=vr0dc8meaabaqaciaacaGaaeqabaqabeGadaaakeaaiiGacuWFbpGCgaqcaaaa@2E83@_*B *_equals -1 in the BRMAs of the telomerase and CD4 datasets. For the telomerase data there are 10 studies; the simulations show that this magnitude of studies will often provide little information about *ρ*_*B*_, causing ρ^
 MathType@MTEF@5@5@+=feaafiart1ev1aaatCvAUfKttLearuWrP9MDH5MBPbIqV92AaeXatLxBI9gBaebbnrfifHhDYfgasaacH8akY=wiFfYdH8Gipec8Eeeu0xXdbba9frFj0=OqFfea0dXdd9vqai=hGuQ8kuc9pgc9s8qqaq=dirpe0xb9q8qiLsFr0=vr0=vr0dc8meaabaqaciaacaGaaeqabaqabeGadaaakeaaiiGacuWFbpGCgaqcaaaa@2E83@_*B *_to often be constrained at -1 or +1 so that the restrictions imposed on **V **are met. For the CD4 data, even though there are five more studies than telomerase, the mean within-study variance for endpoint *j *= 1 is 0.15 and this is large relative to the between-study variation (τ^12
 MathType@MTEF@5@5@+=feaafiart1ev1aaatCvAUfKttLearuWrP9MDH5MBPbIqV92AaeXatLxBI9gBaebbnrfifHhDYfgasaacH8akY=wiFfYdH8Gipec8Eeeu0xXdbba9frFj0=OqFfea0dXdd9vqai=hGuQ8kuc9pgc9s8qqaq=dirpe0xb9q8qiLsFr0=vr0=vr0dc8meaabaqaciaacaGaaeqabaqabeGadaaakeaaiiGacuWFepaDgaqcamaaDaaaleaacqaIXaqmaeaacqaIYaGmaaaaaa@3097@ = 0.048). In such situations where the within-study variation dominates, the simulations again show that ρ^
 MathType@MTEF@5@5@+=feaafiart1ev1aaatCvAUfKttLearuWrP9MDH5MBPbIqV92AaeXatLxBI9gBaebbnrfifHhDYfgasaacH8akY=wiFfYdH8Gipec8Eeeu0xXdbba9frFj0=OqFfea0dXdd9vqai=hGuQ8kuc9pgc9s8qqaq=dirpe0xb9q8qiLsFr0=vr0=vr0dc8meaabaqaciaacaGaaeqabaqabeGadaaakeaaiiGacuWFbpGCgaqcaaaa@2E83@_*B *_will often require truncation at the end of its parameter space to ensure **V **is non-negative definite.

#### Between-study variance estimates

Our simulation results show that the between-study variance estimates were less frequently truncated at zero in the BRMA than the URMA (Table 4). For example, in scenario (ii) with *n *= 5 τ^22
 MathType@MTEF@5@5@+=feaafiart1ev1aaatCvAUfKttLearuWrP9MDH5MBPbIqV92AaeXatLxBI9gBaebbnrfifHhDYfgasaacH8akY=wiFfYdH8Gipec8Eeeu0xXdbba9frFj0=OqFfea0dXdd9vqai=hGuQ8kuc9pgc9s8qqaq=dirpe0xb9q8qiLsFr0=vr0=vr0dc8meaabaqaciaacaGaaeqabaqabeGadaaakeaaiiGacuWFepaDgaqcamaaDaaaleaacqaIYaGmaeaacqaIYaGmaaaaaa@3099@ was zero for 104 of the URMA simulations and none of the BRMA simulations. Furthermore, in those scenarios where the between-study correlation was often +1 or -1 (e.g. scenario (ii) with *n *= 5), the normal BRMA model produces a noticeable upward bias in the between-study variance estimates. For example, in scenario (ii) with *n *= 5 there was an upward bias of 0.024 in τ^12
 MathType@MTEF@5@5@+=feaafiart1ev1aaatCvAUfKttLearuWrP9MDH5MBPbIqV92AaeXatLxBI9gBaebbnrfifHhDYfgasaacH8akY=wiFfYdH8Gipec8Eeeu0xXdbba9frFj0=OqFfea0dXdd9vqai=hGuQ8kuc9pgc9s8qqaq=dirpe0xb9q8qiLsFr0=vr0=vr0dc8meaabaqaciaacaGaaeqabaqabeGadaaakeaaiiGacuWFepaDgaqcamaaDaaaleaacqaIXaqmaeaacqaIYaGmaaaaaa@3097@ and τ^22
 MathType@MTEF@5@5@+=feaafiart1ev1aaatCvAUfKttLearuWrP9MDH5MBPbIqV92AaeXatLxBI9gBaebbnrfifHhDYfgasaacH8akY=wiFfYdH8Gipec8Eeeu0xXdbba9frFj0=OqFfea0dXdd9vqai=hGuQ8kuc9pgc9s8qqaq=dirpe0xb9q8qiLsFr0=vr0=vr0dc8meaabaqaciaacaGaaeqabaqabeGadaaakeaaiiGacuWFepaDgaqcamaaDaaaleaacqaIYaGmaeaacqaIYaGmaaaaaa@3099@, about 10% above their true value. To understand analytically why this occurs, we need to consider that the between-study covariance (*τ*_12_) is formulated by *τ*_12 _= *ρ*_*B *_*τ*_1 _*τ*_2_. Now, if ρ^
 MathType@MTEF@5@5@+=feaafiart1ev1aaatCvAUfKttLearuWrP9MDH5MBPbIqV92AaeXatLxBI9gBaebbnrfifHhDYfgasaacH8akY=wiFfYdH8Gipec8Eeeu0xXdbba9frFj0=OqFfea0dXdd9vqai=hGuQ8kuc9pgc9s8qqaq=dirpe0xb9q8qiLsFr0=vr0=vr0dc8meaabaqaciaacaGaaeqabaqabeGadaaakeaaiiGacuWFbpGCgaqcaaaa@2E83@_*B *_is constrained at -1 or +1, then to obtain the necessary solution for τ^
 MathType@MTEF@5@5@+=feaafiart1ev1aaatCvAUfKttLearuWrP9MDH5MBPbIqV92AaeXatLxBI9gBaebbnrfifHhDYfgasaacH8akY=wiFfYdH8Gipec8Eeeu0xXdbba9frFj0=OqFfea0dXdd9vqai=hGuQ8kuc9pgc9s8qqaq=dirpe0xb9q8qiLsFr0=vr0=vr0dc8meaabaqaciaacaGaaeqabaqabeGadaaakeaaiiGacuWFepaDgaqcaaaa@2E88@_12 _the maximum likelihood estimator can only increase the τ^
 MathType@MTEF@5@5@+=feaafiart1ev1aaatCvAUfKttLearuWrP9MDH5MBPbIqV92AaeXatLxBI9gBaebbnrfifHhDYfgasaacH8akY=wiFfYdH8Gipec8Eeeu0xXdbba9frFj0=OqFfea0dXdd9vqai=hGuQ8kuc9pgc9s8qqaq=dirpe0xb9q8qiLsFr0=vr0=vr0dc8meaabaqaciaacaGaaeqabaqabeGadaaakeaaiiGacuWFepaDgaqcaaaa@2E88@_*j *_s, which do not have an upper bound constraint. Thus the between-study variance estimates are inflated to compensate for the constraint on ρ^
 MathType@MTEF@5@5@+=feaafiart1ev1aaatCvAUfKttLearuWrP9MDH5MBPbIqV92AaeXatLxBI9gBaebbnrfifHhDYfgasaacH8akY=wiFfYdH8Gipec8Eeeu0xXdbba9frFj0=OqFfea0dXdd9vqai=hGuQ8kuc9pgc9s8qqaq=dirpe0xb9q8qiLsFr0=vr0=vr0dc8meaabaqaciaacaGaaeqabaqabeGadaaakeaaiiGacuWFbpGCgaqcaaaa@2E83@_*B*_. This explains why the BRMAs of the telomerase and CD4 data, where ρ^
 MathType@MTEF@5@5@+=feaafiart1ev1aaatCvAUfKttLearuWrP9MDH5MBPbIqV92AaeXatLxBI9gBaebbnrfifHhDYfgasaacH8akY=wiFfYdH8Gipec8Eeeu0xXdbba9frFj0=OqFfea0dXdd9vqai=hGuQ8kuc9pgc9s8qqaq=dirpe0xb9q8qiLsFr0=vr0=vr0dc8meaabaqaciaacaGaaeqabaqabeGadaaakeaaiiGacuWFbpGCgaqcaaaa@2E83@_*B *_was truncated at -1, give τ^
 MathType@MTEF@5@5@+=feaafiart1ev1aaatCvAUfKttLearuWrP9MDH5MBPbIqV92AaeXatLxBI9gBaebbnrfifHhDYfgasaacH8akY=wiFfYdH8Gipec8Eeeu0xXdbba9frFj0=OqFfea0dXdd9vqai=hGuQ8kuc9pgc9s8qqaq=dirpe0xb9q8qiLsFr0=vr0=vr0dc8meaabaqaciaacaGaaeqabaqabeGadaaakeaaiiGacuWFepaDgaqcaaaa@2E88@_*j *_s that are noticeably larger than those from two separate URMAs. Practitioners need to be aware of this issue; however, we do not consider it a major concern as the maximum likelihood estimator for τj2
 MathType@MTEF@5@5@+=feaafiart1ev1aaatCvAUfKttLearuWrP9MDH5MBPbIqV92AaeXatLxBI9gBaebbnrfifHhDYfgasaacH8akY=wiFfYdH8Gipec8Eeeu0xXdbba9frFj0=OqFfea0dXdd9vqai=hGuQ8kuc9pgc9s8qqaq=dirpe0xb9q8qiLsFr0=vr0=vr0dc8meaabaqaciaacaGaaeqabaqabeGadaaakeaaiiGacqWFepaDdaqhaaWcbaGaemOAaOgabaGaeGOmaidaaaaa@30F4@ is still asymptotically unbiased (the bias decreases as the number of studies increases) and the inflation is simply caused by the sensible and necessary constraint on ρ^
 MathType@MTEF@5@5@+=feaafiart1ev1aaatCvAUfKttLearuWrP9MDH5MBPbIqV92AaeXatLxBI9gBaebbnrfifHhDYfgasaacH8akY=wiFfYdH8Gipec8Eeeu0xXdbba9frFj0=OqFfea0dXdd9vqai=hGuQ8kuc9pgc9s8qqaq=dirpe0xb9q8qiLsFr0=vr0=vr0dc8meaabaqaciaacaGaaeqabaqabeGadaaakeaaiiGacuWFbpGCgaqcaaaa@2E83@_*B*_. Furthermore, the inflation is essentially conservative, leading to a larger standard error and mean-square error of pooled estimates as now discussed.

#### Pooled estimates

For all complete and missing data scenarios, the pooled estimates were approximately unbiased for both BRMA and URMA. Even in those scenarios where ρ^
 MathType@MTEF@5@5@+=feaafiart1ev1aaatCvAUfKttLearuWrP9MDH5MBPbIqV92AaeXatLxBI9gBaebbnrfifHhDYfgasaacH8akY=wiFfYdH8Gipec8Eeeu0xXdbba9frFj0=OqFfea0dXdd9vqai=hGuQ8kuc9pgc9s8qqaq=dirpe0xb9q8qiLsFr0=vr0=vr0dc8meaabaqaciaacaGaaeqabaqabeGadaaakeaaiiGacuWFbpGCgaqcaaaa@2E83@_*B *_was often + 1 or -1 it is encouraging that, despite the upward bias in between-study variances, there was no systematic bias in the pooled estimates from the BRMA (Table 4 and Appendix 1 – see Additional file 1). The main affect on the pooled estimates was an inflated standard error and mean-square error. This is a conservative property, but meant that in some complete data scenarios the BRMA performed slightly worse than URMA, despite the utilisation of correlation in the BRMA that might be expected to improve efficiency [10]. For example, in the *n = 5 *results for scenario (iii), where about 56% of simulations gave ρ^
 MathType@MTEF@5@5@+=feaafiart1ev1aaatCvAUfKttLearuWrP9MDH5MBPbIqV92AaeXatLxBI9gBaebbnrfifHhDYfgasaacH8akY=wiFfYdH8Gipec8Eeeu0xXdbba9frFj0=OqFfea0dXdd9vqai=hGuQ8kuc9pgc9s8qqaq=dirpe0xb9q8qiLsFr0=vr0=vr0dc8meaabaqaciaacaGaaeqabaqabeGadaaakeaaiiGacuWFbpGCgaqcaaaa@2E83@_*B *_equal to +1 or -1 and there was an upward bias in between-study variances, the standard error/mean-square error of β^
 MathType@MTEF@5@5@+=feaafiart1ev1aaatCvAUfKttLearuWrP9MDH5MBPbIqV92AaeXatLxBI9gBaebbnrfifHhDYfgasaacH8akY=wiFfYdH8Gipec8Eeeu0xXdbba9frFj0=OqFfea0dXdd9vqai=hGuQ8kuc9pgc9s8qqaq=dirpe0xb9q8qiLsFr0=vr0=vr0dc8meaabaqaciaacaGaaeqabaqabeGadaaakeaaiiGacuWFYoGygaqcaaaa@2E64@_1 _was larger in the BRMA (0.272/0.083) than the URMA (0.268/0.081). This explains the BRMA results for telomerase and CD4, where the standard errors of pooled estimates were larger in the BRMA than the URMA due to the inflated between-study variances.

The coverage of *β*_1 _and *β*_2 _was between 93% and 98% in most scenarios assessed, and was often similar in URMA and BRMA. It is hard, though, to make general conclusions regarding coverage, as those situations where ρ^
 MathType@MTEF@5@5@+=feaafiart1ev1aaatCvAUfKttLearuWrP9MDH5MBPbIqV92AaeXatLxBI9gBaebbnrfifHhDYfgasaacH8akY=wiFfYdH8Gipec8Eeeu0xXdbba9frFj0=OqFfea0dXdd9vqai=hGuQ8kuc9pgc9s8qqaq=dirpe0xb9q8qiLsFr0=vr0=vr0dc8meaabaqaciaacaGaaeqabaqabeGadaaakeaaiiGacuWFbpGCgaqcaaaa@2E83@_*B *_is often +1 or -1 are the same situations where the *t*-distribution with *n*-1 degrees of freedom is a poor approximation to the true sampling distribution. The true degrees of freedom to use here are complex and account for the within-study variances [12]; however this is rarely done in meta-analysis and is beyond the scope of this paper.

#### BRMA versus URMA for estimating the pooled values

For complete data, in most scenarios the BRMA was marginally superior to URMA as the pooled estimates had slightly smaller standard errors and mean-square errors, especially given large correlations (Table 4 and Appendix 1 – see Additional file 1). However, the URMA sometimes performed equally well, and occasionally even better in those scenarios where ρ^
 MathType@MTEF@5@5@+=feaafiart1ev1aaatCvAUfKttLearuWrP9MDH5MBPbIqV92AaeXatLxBI9gBaebbnrfifHhDYfgasaacH8akY=wiFfYdH8Gipec8Eeeu0xXdbba9frFj0=OqFfea0dXdd9vqai=hGuQ8kuc9pgc9s8qqaq=dirpe0xb9q8qiLsFr0=vr0=vr0dc8meaabaqaciaacaGaaeqabaqabeGadaaakeaaiiGacuWFbpGCgaqcaaaa@2E83@_*B *_was often +1 or -1 as discussed above. We also compared the subset of BRMA results where ρ^
 MathType@MTEF@5@5@+=feaafiart1ev1aaatCvAUfKttLearuWrP9MDH5MBPbIqV92AaeXatLxBI9gBaebbnrfifHhDYfgasaacH8akY=wiFfYdH8Gipec8Eeeu0xXdbba9frFj0=OqFfea0dXdd9vqai=hGuQ8kuc9pgc9s8qqaq=dirpe0xb9q8qiLsFr0=vr0=vr0dc8meaabaqaciaacaGaaeqabaqabeGadaaakeaaiiGacuWFbpGCgaqcaaaa@2E83@_*B *_did not equal +1 or -1 with the corresponding URMA results, and again found that BRMA was generally slightly superior to URMA. This finding agrees with previous algebraic results [10], that given complete data there is generally a very small benefit of BRMA over URMA for estimating *β*_1 _and *β*_2 _themselves. Our focus here is on the individual pooled estimates, but we note that there are also broader reasons why a BRMA may be preferred over URMA for complete data. These are summarised in the Discussion to ensure a more complete picture for practitioners considering BRMA.

For the missing data simulations, the pooled estimate for endpoint *j = 2 *was of particular interest because of the missing data for this endpoint. Encouragingly, the mean-square error and mean standard error of β^
 MathType@MTEF@5@5@+=feaafiart1ev1aaatCvAUfKttLearuWrP9MDH5MBPbIqV92AaeXatLxBI9gBaebbnrfifHhDYfgasaacH8akY=wiFfYdH8Gipec8Eeeu0xXdbba9frFj0=OqFfea0dXdd9vqai=hGuQ8kuc9pgc9s8qqaq=dirpe0xb9q8qiLsFr0=vr0=vr0dc8meaabaqaciaacaGaaeqabaqabeGadaaakeaaiiGacuWFYoGygaqcaaaa@2E64@_2 _were much smaller in the BRMA than the URMA, although the coverage was comparable (Table 4 and Appendix 1 – see Additional file 1). For example, in the *n = 10 *simulations of scenario (xi) the mean standard error was 0.225 in the BRMA compared to 0.262 in the URMA, and the MSE was 0.0708 in the BRMA compared to 0.0921 in the URMA. The reduction in standard error and MSE was larger when both the within- and between-study correlations were high. Even when *ρ*_*Wi *_was zero there was still a reasonable benefit if *ρ*_*B *_was high; for example, in the *n = 10 *simulations of scenario (viii) the mean standard error of β^
 MathType@MTEF@5@5@+=feaafiart1ev1aaatCvAUfKttLearuWrP9MDH5MBPbIqV92AaeXatLxBI9gBaebbnrfifHhDYfgasaacH8akY=wiFfYdH8Gipec8Eeeu0xXdbba9frFj0=OqFfea0dXdd9vqai=hGuQ8kuc9pgc9s8qqaq=dirpe0xb9q8qiLsFr0=vr0=vr0dc8meaabaqaciaacaGaaeqabaqabeGadaaakeaaiiGacuWFYoGygaqcaaaa@2E64@_2 _was 0.174 in the BRMA compared to 0.209 in the URMA. This finding agrees with algebraic work regarding the benefits of BRMA for when there are data missing at random [10]. Practitioners should again consider this benefit alongside the other broader reasons for using BRMA rather than URMA (see Discussion).

### Extended simulations of the normal BRMA model

In our above simulations of the normal BRMA model we used non-negative within- and between-study correlations; however, in reality negative correlations may arise as in the telomerase and CD4 examples. Also, our simulations took the within-study correlations to be the same in each study, while in reality their value may vary. Further simulations were thus performed to assess negative correlation and discrepant within-study correlations. These gave findings consistent with those identified previously (Appendix 2 – see Additional file 2); the BRMA was still beneficial over URMA for estimating the pooled endpoints, and where the between-study correlation estimate was often +1 or -1 there was again an upward bias in the BRMA between-study variance estimates.

For simplicity, in all our simulations si12
 MathType@MTEF@5@5@+=feaafiart1ev1aaatCvAUfKttLearuWrP9MDH5MBPbIqV92AaeXatLxBI9gBaebbnrfifHhDYfgasaacH8akY=wiFfYdH8Gipec8Eeeu0xXdbba9frFj0=OqFfea0dXdd9vqai=hGuQ8kuc9pgc9s8qqaq=dirpe0xb9q8qiLsFr0=vr0=vr0dc8meaabaqaciaacaGaaeqabaqabeGadaaakeaacqWGZbWCdaqhaaWcbaGaemyAaKMaeGymaedabaGaeGOmaidaaaaa@3185@ and si22
 MathType@MTEF@5@5@+=feaafiart1ev1aaatCvAUfKttLearuWrP9MDH5MBPbIqV92AaeXatLxBI9gBaebbnrfifHhDYfgasaacH8akY=wiFfYdH8Gipec8Eeeu0xXdbba9frFj0=OqFfea0dXdd9vqai=hGuQ8kuc9pgc9s8qqaq=dirpe0xb9q8qiLsFr0=vr0=vr0dc8meaabaqaciaacaGaaeqabaqabeGadaaakeaacqWGZbWCdaqhaaWcbaGaemyAaKMaeGOmaidabaGaeGOmaidaaaaa@3187@ were generated independently but in reality they are likely to be correlated due to the sample size being similar for both endpoints. We also generated the sij2
 MathType@MTEF@5@5@+=feaafiart1ev1aaatCvAUfKttLearuWrP9MDH5MBPbIqV92AaeXatLxBI9gBaebbnrfifHhDYfgasaacH8akY=wiFfYdH8Gipec8Eeeu0xXdbba9frFj0=OqFfea0dXdd9vqai=hGuQ8kuc9pgc9s8qqaq=dirpe0xb9q8qiLsFr0=vr0=vr0dc8meaabaqaciaacaGaaeqabaqabeGadaaakeaacqWGZbWCdaqhaaWcbaGaemyAaKMaemOAaOgabaGaeGOmaidaaaaa@31F2@ s independent to the *Y*_*ij *_s, yet in many situations, such as the synthesis of log-odds ratios, the size of sij2
 MathType@MTEF@5@5@+=feaafiart1ev1aaatCvAUfKttLearuWrP9MDH5MBPbIqV92AaeXatLxBI9gBaebbnrfifHhDYfgasaacH8akY=wiFfYdH8Gipec8Eeeu0xXdbba9frFj0=OqFfea0dXdd9vqai=hGuQ8kuc9pgc9s8qqaq=dirpe0xb9q8qiLsFr0=vr0=vr0dc8meaabaqaciaacaGaaeqabaqabeGadaaakeaacqWGZbWCdaqhaaWcbaGaemyAaKMaemOAaOgabaGaeGOmaidaaaaa@31F2@ may be related to the size of *Y*_*ij*_. To address this, we also performed further simulations where we firstly generated individual binary data for diagnostic studies, using simulation code kindly provided by Chu and Cole [12]. From this realistic raw data we then calculated the *Y*_*ij *_s and their sij2
 MathType@MTEF@5@5@+=feaafiart1ev1aaatCvAUfKttLearuWrP9MDH5MBPbIqV92AaeXatLxBI9gBaebbnrfifHhDYfgasaacH8akY=wiFfYdH8Gipec8Eeeu0xXdbba9frFj0=OqFfea0dXdd9vqai=hGuQ8kuc9pgc9s8qqaq=dirpe0xb9q8qiLsFr0=vr0=vr0dc8meaabaqaciaacaGaaeqabaqabeGadaaakeaacqWGZbWCdaqhaaWcbaGaemyAaKMaemOAaOgabaGaeGOmaidaaaaa@31F2@ s, before then fitting the normal BRMA model as before. The results again show that the between-study correlation estimate is often +1 or -1 and the BRMA is still preferable to URMA, with improved mean-square error, coverage and, especially, bias of estimates (Table 5). However, the results also revealed some severe limitations of a normal model for meta-analysis of binary data, as now discussed.

**Table 5:** simulation results for meta-analysis of proportions

**Meta-analysis model**	***n***	**No. of the 1000 simulations that converged**	**Bias of mean **β^ MathType@MTEF@5@5@+=feaafiart1ev1aaatCvAUfKttLearuWrP9MDH5MBPbIqV92AaeXatLxBI9gBaebbnrfifHhDYfgasaacH8akY=wiFfYdH8Gipec8Eeeu0xXdbba9frFj0=OqFfea0dXdd9vqai=hGuQ8kuc9pgc9s8qqaq=dirpe0xb9q8qiLsFr0=vr0=vr0dc8meaabaqaciaacaGaaeqabaqabeGadaaakeaaiiGacuWFYoGygaqcaaaa@2E64@_1_	**Mean s.e. of **β^ MathType@MTEF@5@5@+=feaafiart1ev1aaatCvAUfKttLearuWrP9MDH5MBPbIqV92AaeXatLxBI9gBaebbnrfifHhDYfgasaacH8akY=wiFfYdH8Gipec8Eeeu0xXdbba9frFj0=OqFfea0dXdd9vqai=hGuQ8kuc9pgc9s8qqaq=dirpe0xb9q8qiLsFr0=vr0=vr0dc8meaabaqaciaacaGaaeqabaqabeGadaaakeaaiiGacuWFYoGygaqcaaaa@2E64@_1_	**MSE of **β^ MathType@MTEF@5@5@+=feaafiart1ev1aaatCvAUfKttLearuWrP9MDH5MBPbIqV92AaeXatLxBI9gBaebbnrfifHhDYfgasaacH8akY=wiFfYdH8Gipec8Eeeu0xXdbba9frFj0=OqFfea0dXdd9vqai=hGuQ8kuc9pgc9s8qqaq=dirpe0xb9q8qiLsFr0=vr0=vr0dc8meaabaqaciaacaGaaeqabaqabeGadaaakeaaiiGacuWFYoGygaqcaaaa@2E64@_1_	**Coverage of the 95% CIs for **β^ MathType@MTEF@5@5@+=feaafiart1ev1aaatCvAUfKttLearuWrP9MDH5MBPbIqV92AaeXatLxBI9gBaebbnrfifHhDYfgasaacH8akY=wiFfYdH8Gipec8Eeeu0xXdbba9frFj0=OqFfea0dXdd9vqai=hGuQ8kuc9pgc9s8qqaq=dirpe0xb9q8qiLsFr0=vr0=vr0dc8meaabaqaciaacaGaaeqabaqabeGadaaakeaaiiGacuWFYoGygaqcaaaa@2E64@_1_	**Bias of mean **β^ MathType@MTEF@5@5@+=feaafiart1ev1aaatCvAUfKttLearuWrP9MDH5MBPbIqV92AaeXatLxBI9gBaebbnrfifHhDYfgasaacH8akY=wiFfYdH8Gipec8Eeeu0xXdbba9frFj0=OqFfea0dXdd9vqai=hGuQ8kuc9pgc9s8qqaq=dirpe0xb9q8qiLsFr0=vr0=vr0dc8meaabaqaciaacaGaaeqabaqabeGadaaakeaaiiGacuWFYoGygaqcaaaa@2E64@_2_	**Mean s.e. of **β^ MathType@MTEF@5@5@+=feaafiart1ev1aaatCvAUfKttLearuWrP9MDH5MBPbIqV92AaeXatLxBI9gBaebbnrfifHhDYfgasaacH8akY=wiFfYdH8Gipec8Eeeu0xXdbba9frFj0=OqFfea0dXdd9vqai=hGuQ8kuc9pgc9s8qqaq=dirpe0xb9q8qiLsFr0=vr0=vr0dc8meaabaqaciaacaGaaeqabaqabeGadaaakeaaiiGacuWFYoGygaqcaaaa@2E64@_2_	**MSE of **β^ MathType@MTEF@5@5@+=feaafiart1ev1aaatCvAUfKttLearuWrP9MDH5MBPbIqV92AaeXatLxBI9gBaebbnrfifHhDYfgasaacH8akY=wiFfYdH8Gipec8Eeeu0xXdbba9frFj0=OqFfea0dXdd9vqai=hGuQ8kuc9pgc9s8qqaq=dirpe0xb9q8qiLsFr0=vr0=vr0dc8meaabaqaciaacaGaaeqabaqabeGadaaakeaaiiGacuWFYoGygaqcaaaa@2E64@_2_	**Coverage of the 95% CIs for **β^ MathType@MTEF@5@5@+=feaafiart1ev1aaatCvAUfKttLearuWrP9MDH5MBPbIqV92AaeXatLxBI9gBaebbnrfifHhDYfgasaacH8akY=wiFfYdH8Gipec8Eeeu0xXdbba9frFj0=OqFfea0dXdd9vqai=hGuQ8kuc9pgc9s8qqaq=dirpe0xb9q8qiLsFr0=vr0=vr0dc8meaabaqaciaacaGaaeqabaqabeGadaaakeaaiiGacuWFYoGygaqcaaaa@2E64@_2_	**Bias of mean **τ^12 MathType@MTEF@5@5@+=feaafiart1ev1aaatCvAUfKttLearuWrP9MDH5MBPbIqV92AaeXatLxBI9gBaebbnrfifHhDYfgasaacH8akY=wiFfYdH8Gipec8Eeeu0xXdbba9frFj0=OqFfea0dXdd9vqai=hGuQ8kuc9pgc9s8qqaq=dirpe0xb9q8qiLsFr0=vr0=vr0dc8meaabaqaciaacaGaaeqabaqabeGadaaakeaaiiGacuWFepaDgaqcamaaDaaaleaacqaIXaqmaeaacqaIYaGmaaaaaa@3097@**(no. of **τ^12 MathType@MTEF@5@5@+=feaafiart1ev1aaatCvAUfKttLearuWrP9MDH5MBPbIqV92AaeXatLxBI9gBaebbnrfifHhDYfgasaacH8akY=wiFfYdH8Gipec8Eeeu0xXdbba9frFj0=OqFfea0dXdd9vqai=hGuQ8kuc9pgc9s8qqaq=dirpe0xb9q8qiLsFr0=vr0=vr0dc8meaabaqaciaacaGaaeqabaqabeGadaaakeaaiiGacuWFepaDgaqcamaaDaaaleaacqaIXaqmaeaacqaIYaGmaaaaaa@3097@**= 0)**	**Bias of mean **τ^22 MathType@MTEF@5@5@+=feaafiart1ev1aaatCvAUfKttLearuWrP9MDH5MBPbIqV92AaeXatLxBI9gBaebbnrfifHhDYfgasaacH8akY=wiFfYdH8Gipec8Eeeu0xXdbba9frFj0=OqFfea0dXdd9vqai=hGuQ8kuc9pgc9s8qqaq=dirpe0xb9q8qiLsFr0=vr0=vr0dc8meaabaqaciaacaGaaeqabaqabeGadaaakeaaiiGacuWFepaDgaqcamaaDaaaleaacqaIYaGmaeaacqaIYaGmaaaaaa@3099@**(no. of **τ^22 MathType@MTEF@5@5@+=feaafiart1ev1aaatCvAUfKttLearuWrP9MDH5MBPbIqV92AaeXatLxBI9gBaebbnrfifHhDYfgasaacH8akY=wiFfYdH8Gipec8Eeeu0xXdbba9frFj0=OqFfea0dXdd9vqai=hGuQ8kuc9pgc9s8qqaq=dirpe0xb9q8qiLsFr0=vr0=vr0dc8meaabaqaciaacaGaaeqabaqabeGadaaakeaaiiGacuWFepaDgaqcamaaDaaaleaacqaIYaGmaeaacqaIYaGmaaaaaa@3099@**= 0)**	**Bias of mean **ρ^ MathType@MTEF@5@5@+=feaafiart1ev1aaatCvAUfKttLearuWrP9MDH5MBPbIqV92AaeXatLxBI9gBaebbnrfifHhDYfgasaacH8akY=wiFfYdH8Gipec8Eeeu0xXdbba9frFj0=OqFfea0dXdd9vqai=hGuQ8kuc9pgc9s8qqaq=dirpe0xb9q8qiLsFr0=vr0=vr0dc8meaabaqaciaacaGaaeqabaqabeGadaaakeaaiiGacuWFbpGCgaqcaaaa@2E83@_*B*_	**% of **ρ^ MathType@MTEF@5@5@+=feaafiart1ev1aaatCvAUfKttLearuWrP9MDH5MBPbIqV92AaeXatLxBI9gBaebbnrfifHhDYfgasaacH8akY=wiFfYdH8Gipec8Eeeu0xXdbba9frFj0=OqFfea0dXdd9vqai=hGuQ8kuc9pgc9s8qqaq=dirpe0xb9q8qiLsFr0=vr0=vr0dc8meaabaqaciaacaGaaeqabaqabeGadaaakeaaiiGacuWFbpGCgaqcaaaa@2E83@_*B *_**= -1**	**% of **ρ^ MathType@MTEF@5@5@+=feaafiart1ev1aaatCvAUfKttLearuWrP9MDH5MBPbIqV92AaeXatLxBI9gBaebbnrfifHhDYfgasaacH8akY=wiFfYdH8Gipec8Eeeu0xXdbba9frFj0=OqFfea0dXdd9vqai=hGuQ8kuc9pgc9s8qqaq=dirpe0xb9q8qiLsFr0=vr0=vr0dc8meaabaqaciaacaGaaeqabaqabeGadaaakeaaiiGacuWFbpGCgaqcaaaa@2E83@_*B *_**= 1**
**Complete data – we set 25 true positives and 25 true negatives in each study, with the pooled logit-sensitivity and pooled logit-specificity set at 1.386 (i.e. a sensitivity and specificity of 0.8). The between-study correlation was set as -0.8, and the between-study variation was set as 1 for both sensitivity and specificity. Simulations were performed as in Chu and Cole [12].**															
Normal URMA	10	995	-0.152	0.311	0.120	93.1%	-0.174	0.310	0.119	93.2%	-0.313 (17)	-0.309 (5)	-	-	-
Normal BRMA	10	995	-0.124	0.318	0.118	93.8%	-0.144	0.317	0.115	94.5%	-0.245 (7)	-0.245 (2)	0.067	53.7%	0%
Generalised URMA	10	1000	-0.013	0.330	0.124	93.8%	-0.037	0.328	0.116	94.0%	-0.186 (20)	-0.189 (21)	-	-	-
Generalised URMA	10	603*	-0.012	0.339	0.119	94.9%	-0.030	0.342	0.118	95.8%	-0.136 (0)	-0.114 (0)	-	-	-
Generalised BRMA	10	603	-0.009	0.339	0.119	95.4%	-0.029	0.341	0.116	96.2%	-0.134 (0)	-0.113 (0)	-0.084	0%	0%
Normal URMA	50	1000	-0.175	0.141	0.049	77.2%	-0.182	0.141	0.050	75.5%	-0.337 (0)	-0.335 (0)	-	-	-
Normal BRMA	50	1000	-0.151	0.142	0.042	81.4%	-0.157	0.143	0.043	81.3%	-0.285 (0)	-0.282 (0)	0.087	17.0%	0%
Generalised URMA	50	1000	-0.018	0.157	0.024	95.1%	-0.026	0.157	0.023	96.1%	-0.091 (0)	-0.084 (0)	-	-	-
Generalised URMA	50	973*	-0.019	0.157	0.024	95.1%	-0.022	0.157	0.023	96.1%	-0.087 (0)	-0.080 (0)	-	-	-
Generalised BRMA	50	973	-0.016	0.157	0.024	96.0%	-0.020	0.158	0.023	96.2%	-0.078 (0)	-0.071 (0)	0.019	0%	0%

MSE = mean-square-error, *n *= number of studies in each meta-analysis, CIs = confidence intervals, s.e. = standard error;

Restricted maximum likelihood estimation was used for the normal models, whereas maximum likelihood estimation was used for the generalised models.

* These were a subset of the 1000 URMA simulations that converged and were the same ones that converged in the equivalent BRMA; this helps fairly compare the URMA and BRMA results.

### A generalised model for BRMA of proportions

So far in this paper we have modelled the summary statistics across studies, i.e. the *Y*_*ij *_s, and assumed they are normally distributed. Indeed, in our main simulations we generated the *Y*_*ij *_s directly from the normal BRMA model of equation (1); thus, our conclusions are only valid for *Y*_*ij *_s that can truly be assumed normally distributed. This normality assumption is common in the meta-analysis field, and will often be suitable (see Discussion). However, in our two motivating examples it is more plausible for the CD4 data than the telomerase data as the latter involves a meta-analysis of proportions, for which the normality assumption is not appropriate when some studies have a small number of patients or the proportions are close to 0 or 1. For this reason recent articles [12] suggest that, rather than modelling the logit-proportions using the normal distribution, one should directly model the binary data using a binomial distribution. This approach also avoids the use of ad hoc continuity corrections in those studies which have zero cells. In terms of diagnostic studies, this generalised model for BRMA of sensitivity and specificity can be written as follows [12]:

no. testing positive_*i*_~*Binomial *(total no. true positives_*i*_, sensitivity_*i*_) logit(sensitivity_*i*_) = *β*_1 _+ *u*_1_

no. testing negative_*i*_~*Binomial *(total no. true negatives_*i*_, specificity_*i*_) logit(specificity_*i*_) = *β*_2 _+ *u*_2_

(u1u2)~N[(00),Ω],Ω=(τ12τ1τ2ρBτ1τ2ρBτ22)     (3)
 MathType@MTEF@5@5@+=feaafiart1ev1aaatCvAUfKttLearuWrP9MDH5MBPbIqV92AaeXatLxBI9gBaebbnrfifHhDYfgasaacH8akY=wiFfYdH8Gipec8Eeeu0xXdbba9frFj0=OqFfea0dXdd9vqai=hGuQ8kuc9pgc9s8qqaq=dirpe0xb9q8qiLsFr0=vr0=vr0dc8meaabaqaciaacaGaaeqabaqabeGadaaakeaafaqabeqacaaabaWaaeWaaeaafaqabeGabaaabaGaemyDau3aaSbaaSqaaiabigdaXaqabaaakeaacqWG1bqDdaWgaaWcbaGaeGOmaidabeaaaaaakiaawIcacaGLPaaacqGG+bGFcqWGobGtdaWadaqaamaabmaabaqbaeqabiqaaaqaaiabicdaWaqaaiabicdaWaaaaiaawIcacaGLPaaacqGGSaaliiqacqWFPoWvaiaawUfacaGLDbaacqGGSaalaeaacqWFPoWvcqGH9aqpdaqadaqaauaabeqaciaaaeaaiiGacqGFepaDdaqhaaWcbaGaeGymaedabaGaeGOmaidaaaGcbaGae4hXdq3aaSbaaSqaaiabigdaXaqabaGccqGFepaDdaWgaaWcbaGaeGOmaidabeaakiab+f8aYnaaBaaaleaacqWGcbGqaeqaaaGcbaGae4hXdq3aaSbaaSqaaiabigdaXaqabaGccqGFepaDdaWgaaWcbaGaeGOmaidabeaakiab+f8aYnaaBaaaleaacqWGcbGqaeqaaaGcbaGae4hXdq3aa0baaSqaaiabikdaYaqaaiabikdaYaaaaaaakiaawIcacaGLPaaaaaGaaCzcaiaaxMaadaqadaqaaiabiodaZaGaayjkaiaawMcaaaaa@601B@

Equation (3) can be fitted using maximum likelihood estimation in SAS NLMIXED. Chu and Cole [12] show that where the true sensitivity and specificity are large, this generalised BRMA model produces close to unbiased pooled and between-study correlation estimates, whereas the general normal BRMA model produces somewhat biased estimates. This can also be seen in our simulations results for meta-analysis of proportions in Table 5. The mean-square error, coverage, and, most noticeably, bias of estimates are far superior in the generalised BRMA than the normal BRMA. Furthermore, the generalised BRMA is also marginally superior in terms of bias to two separate generalised URMAs (i.e. equation (3) where *ρ*_*B *_= 0), emphasising that the BRMA is also beneficial over URMA in the generalised model framework. Note though that, although it is the best method, the generalised BRMA model is itself not without bias (Table 5); to rectify this, extension to REML or other estimation techniques is potentially important.

To conclude our research we applied the generalised BRMA model to the telomerase data. Unfortunately the model would not converge appropriately; different starting values all produced a between-study correlation estimate of -1 but gave markedly different parameter estimates and caused spurious standard errors. For example, for one set of starting values the model gave the standard error of β^
 MathType@MTEF@5@5@+=feaafiart1ev1aaatCvAUfKttLearuWrP9MDH5MBPbIqV92AaeXatLxBI9gBaebbnrfifHhDYfgasaacH8akY=wiFfYdH8Gipec8Eeeu0xXdbba9frFj0=OqFfea0dXdd9vqai=hGuQ8kuc9pgc9s8qqaq=dirpe0xb9q8qiLsFr0=vr0=vr0dc8meaabaqaciaacaGaaeqabaqabeGadaaakeaaiiGacuWFYoGygaqcaaaa@2E64@_1 _as 30.5, whereas for another set the standard error was close to zero. Indeed SAS provides the following warning: 'the final Hessian matrix is not positive definite, and therefore the estimated covariance matrix is not full rank and may be unreliable'. The problem here is again due to the between-study correlation of estimate -1 in **Ω**, as this causes the determinant of Ω^
 MathType@MTEF@5@5@+=feaafiart1ev1aaatCvAUfKttLearuWrP9MDH5MBPbIqV92AaeXatLxBI9gBaebbnrfifHhDYfgasaacH8akY=wiFfYdH8Gipec8Eeeu0xXdbba9frFj0=OqFfea0dXdd9vqai=hGuQ8kuc9pgc9s8qqaq=dirpe0xb9q8qiLsFr0=vr0=vr0dc8meaabaqaciaacaGaaeqabaqabeGadaaakeaaiiqacuWFPoWvgaqcaaaa@2E4F@ to be zero. This has greater implications in equation (3) than for the normal BRMA model of equation (1). The maximum likelihood estimator for equation (1) involves the determinant of **δ**_**i **_+ Ω^
 MathType@MTEF@5@5@+=feaafiart1ev1aaatCvAUfKttLearuWrP9MDH5MBPbIqV92AaeXatLxBI9gBaebbnrfifHhDYfgasaacH8akY=wiFfYdH8Gipec8Eeeu0xXdbba9frFj0=OqFfea0dXdd9vqai=hGuQ8kuc9pgc9s8qqaq=dirpe0xb9q8qiLsFr0=vr0=vr0dc8meaabaqaciaacaGaaeqabaqabeGadaaakeaaiiqacuWFPoWvgaqcaaaa@2E4F@ in each study, which will not be zero unless the within-study correlations are also +1 or -1. However, in equation (3) the maximum likelihood estimator involves the determinant of Ω^
 MathType@MTEF@5@5@+=feaafiart1ev1aaatCvAUfKttLearuWrP9MDH5MBPbIqV92AaeXatLxBI9gBaebbnrfifHhDYfgasaacH8akY=wiFfYdH8Gipec8Eeeu0xXdbba9frFj0=OqFfea0dXdd9vqai=hGuQ8kuc9pgc9s8qqaq=dirpe0xb9q8qiLsFr0=vr0=vr0dc8meaabaqaciaacaGaaeqabaqabeGadaaakeaaiiqacuWFPoWvgaqcaaaa@2E4F@ itself, which causes problems akin to dividing by zero, which is why spurious estimates and standard errors are produced. It is clear that there is simply little information to estimate the between-study correlation for the telomerase dataset, due to the small number of studies. This issue is also evident in our *n = 10 *simulations of the generalised BRMA model (Table 5), where 397 of the 1000 simulations did not converge appropriately. In such situations where estimating *ρ*_*B *_is difficult, application of two generalised URMAs may be the most appropriate option available (Table 3), although specifically for diagnostic studies other methods may also be valuable [21].

## Discussion

Multivariate meta-analysis models are increasingly used to synthesise multiple, correlated endpoints of interest, especially in studies of diagnosis [5,21] and surrogate outcomes [7,8]. The Campbell Collaboration suggests that meta-analysts 'should not ignore the dependence among study outcomes'; however, they also note that 'the consequences of accounting for (modelling) dependence or ignoring it are not well understood' [22]. To therefore aid practitioners considering the approach, in this paper we have examined two models for BRMA and compared them to separate univariate syntheses, the traditional approach. We now discuss the main conclusions from our work and suggest future research priorities.

### The general normal model for BRMA

A normal meta-analysis model is appropriate when the *Y*_*ij *_s can be assumed normally distributed; this assumption is commonly used, for example where the *Y*_*ij *_s are log-odds ratios [15], log-hazard ratios [10], mean differences [1] and log-event rates [11].

#### Between-study covariance parameters

It is clear from our work that maximum likelihood estimation of a normal random-effects meta-analysis model will often truncate the between-study covariance matrix, **Ω**, on the boundary of its parameter space. For URMA this is observed by a between-study variance estimate of zero, whilst in BRMA it is more likely observed by a between-study correlation of +1 or -1. Practitioners are likely to be familiar with the concept of zero variance, but are perhaps less likely to appreciate why a correlation is estimated at unity. However, both arise for the same reason, namely that **Ω **must be a non-negative definite matrix such that τj2
 MathType@MTEF@5@5@+=feaafiart1ev1aaatCvAUfKttLearuWrP9MDH5MBPbIqV92AaeXatLxBI9gBaebbnrfifHhDYfgasaacH8akY=wiFfYdH8Gipec8Eeeu0xXdbba9frFj0=OqFfea0dXdd9vqai=hGuQ8kuc9pgc9s8qqaq=dirpe0xb9q8qiLsFr0=vr0=vr0dc8meaabaqaciaacaGaaeqabaqabeGadaaakeaaiiGacqWFepaDdaqhaaWcbaGaemOAaOgabaGaeGOmaidaaaaa@30F4@ is not < 0 and *ρ*_*B *_is not > +1 or < -1. Our simulations show that, especially when the number of studies is small and/or the within-study variance is large relative to the between-study variance, such truncation is often necessary to ensure the sensible restrictions are met. In the normal BRMA, we have also shown that a consequence of ρ^
 MathType@MTEF@5@5@+=feaafiart1ev1aaatCvAUfKttLearuWrP9MDH5MBPbIqV92AaeXatLxBI9gBaebbnrfifHhDYfgasaacH8akY=wiFfYdH8Gipec8Eeeu0xXdbba9frFj0=OqFfea0dXdd9vqai=hGuQ8kuc9pgc9s8qqaq=dirpe0xb9q8qiLsFr0=vr0=vr0dc8meaabaqaciaacaGaaeqabaqabeGadaaakeaaiiGacuWFbpGCgaqcaaaa@2E83@_*B *_being truncated at +1 or -1 is an upward bias in between-study variance estimates, which are inflated upwards to compensate for the restriction on ρ^
 MathType@MTEF@5@5@+=feaafiart1ev1aaatCvAUfKttLearuWrP9MDH5MBPbIqV92AaeXatLxBI9gBaebbnrfifHhDYfgasaacH8akY=wiFfYdH8Gipec8Eeeu0xXdbba9frFj0=OqFfea0dXdd9vqai=hGuQ8kuc9pgc9s8qqaq=dirpe0xb9q8qiLsFr0=vr0=vr0dc8meaabaqaciaacaGaaeqabaqabeGadaaakeaaiiGacuWFbpGCgaqcaaaa@2E83@_*B*_. Practitioners should not, though, be overly concerned by this. We have shown it does not cause any systematic bias in the pooled estimates from BRMA, and it leads to conservative standard errors and mean-square errors.

#### The benefits over URMA for the pooled estimates

Our simulation results highlight that a normal model for BRMA is preferable to two separate URMAs for estimating the pooled endpoints, and our results are consistent with previous findings that show how the inclusion of correlation allows the 'borrowing of strength' across endpoints [1,10,19]. We thus recommend practitioners use a BRMA rather than two separate URMAs where possible. In particular, when some data are missing at random the BRMA is likely to produce a much smaller standard error and mean-square error of pooled estimates than URMA, even for moderate correlations. Riley et al. [10] give an applied example that shows this. For complete data, practitioners should not expect to see much gain in statistical efficiency over URMA; the mean-square error and standard error of pooled estimates are generally only marginally smaller in BRMA than URMA, and on the occasion of ρ^
 MathType@MTEF@5@5@+=feaafiart1ev1aaatCvAUfKttLearuWrP9MDH5MBPbIqV92AaeXatLxBI9gBaebbnrfifHhDYfgasaacH8akY=wiFfYdH8Gipec8Eeeu0xXdbba9frFj0=OqFfea0dXdd9vqai=hGuQ8kuc9pgc9s8qqaq=dirpe0xb9q8qiLsFr0=vr0=vr0dc8meaabaqaciaacaGaaeqabaqabeGadaaakeaaiiGacuWFbpGCgaqcaaaa@2E83@_*B *_= +1 or -1 they may even be slightly worse in BRMA (due to the inflated between-study variances, as in the telomerase and CD4 examples). However, there are broader reasons why BRMA may still be preferable in this situation (see below).

### The generalised model for BRMA

In equation (3) we extended our work to a generalised BRMA model for meta-analysis of two proportions. For synthesis of two proportions, like sensitivity and specificity, this approach is preferable to the general normal BRMA model (Table 5) because the normality assumption breaks down when the proportions are close to 0 or 1 and when there are small patient numbers [12]. It also avoids the use of ad-hoc continuity corrections when there are zero cells in some studies. Practitioners synthesising diagnostic studies are thus encouraged to use the generalised BRMA model, rather than the normal model or indeed two separate generalised URMAs. However, they should also be aware that a between-study correlation estimate of +1 or -1 in the generalised BRMA model is likely to be associated with non-convergence and unstable pooled estimates, as discussed for the telomerase data. In such situations there may be little information to estimate the correlation, and so practitioners may wish to consider other methods for synthesising diagnostic studies, such as the hierarchical summary receiver operating characteristic (HSROC) method [21]; if this is also not possible then the best option may be a generalised URMA for sensitivity and specificity separately (Hamza et al., *personal communication*).

### The broader benefits of BRMA

Our simulations focused mainly on the benefits of BRMA over URMA for estimating the pooled endpoints. However, there are also broader reasons why a BRMA may be preferable to URMA, for either complete or missing data. For example, BRMA allows one to describe the bivariate relationship between endpoints [4,5], model, test or make predictions from their association [7], and estimate some function of the two pooled endpoints, like *β*_1 _- *β*_2 _[10], *β*_1 _+ *β*_2 _[5], or *β*_1_/*β*_2 _[6]. For instance, for the telomerase data, a BRMA enables a single framework to estimate the pooled sensitivity, pooled specificity, and the pooled diagnostic odds ratio (exp(*β*_1 _+ *β*_2_)) [21]. Furthermore, Reitsma et al. [5] show that a BRMA of diagnostic studies enables the correlation between pooled endpoints to be estimated, which allows one to measure the shape of their bivariate relation and construct confidence ellipses. It also allows calculation of the conditional variance in one parameter given a fixed value of the other parameter, and allows drawing of the summary ROC curve. In terms of the CD4 data, the estimated correlation between pooled endpoints from a BRMA enables one to predict the time of onset of AIDS or death from a future patient's CD4 level. Thus the BRMA can help establish whether CD4 should be used as a surrogate of disease-free survival [7,8], and further research of multivariate meta-analysis in this context is recommended. A BRMA may also be extended to a bivariate meta-regression by including additional study-level covariates that explain the between-study heterogeneity. For example, one may wish to include a covariate for study quality in meta-analysis of diagnostic studies [23]. Berkey et al [1] show that a bivariate meta-regression is more efficient than separate univariate meta-regressions for assessing such study-level covariates, again due to the inclusion of correlation.

### Further research suggestions

For the general normal BRMA model, the role of estimation techniques other than REML would be interesting to consider, especially as other potentially better options have just been proposed [24]. For the generalised BRMA model, SAS NLMIXED currently only allows maximum likelihood estimation and so extension to REML is required, especially as the maximum likelihood estimates are not without bias (Table 5). Further research of multivariate meta-analysis within a Bayesian framework is also potentially important, as it would enable the incorporation of prior knowledge about the parameters, which may be valuable when the number of studies is small [15,25]. It would also allow the uncertainty of the si12
 MathType@MTEF@5@5@+=feaafiart1ev1aaatCvAUfKttLearuWrP9MDH5MBPbIqV92AaeXatLxBI9gBaebbnrfifHhDYfgasaacH8akY=wiFfYdH8Gipec8Eeeu0xXdbba9frFj0=OqFfea0dXdd9vqai=hGuQ8kuc9pgc9s8qqaq=dirpe0xb9q8qiLsFr0=vr0=vr0dc8meaabaqaciaacaGaaeqabaqabeGadaaakeaacqWGZbWCdaqhaaWcbaGaemyAaKMaeGymaedabaGaeGOmaidaaaaa@3185@ s, si22
 MathType@MTEF@5@5@+=feaafiart1ev1aaatCvAUfKttLearuWrP9MDH5MBPbIqV92AaeXatLxBI9gBaebbnrfifHhDYfgasaacH8akY=wiFfYdH8Gipec8Eeeu0xXdbba9frFj0=OqFfea0dXdd9vqai=hGuQ8kuc9pgc9s8qqaq=dirpe0xb9q8qiLsFr0=vr0=vr0dc8meaabaqaciaacaGaaeqabaqabeGadaaakeaacqWGZbWCdaqhaaWcbaGaemyAaKMaeGOmaidabaGaeGOmaidaaaaa@3187@ s and *ρ*_*Wi *_s to be taken into account, as in practice they will only be estimates themselves as mentioned by Daniels and Hughes [7]. Further assessment of the role of the within-study correlations is also required, in particular what should we do when they are non-zero but unavailable? For the meta-analysis of surrogate endpoints it has been suggested that the within-study correlations are likely to be small (between 0 and 0.2) and can plausibly be considered constant across studies [7], or even zero [26]. However, this is not necessarily true in other fields; for example, in a multivariate meta-analysis of longitudinal data the within-study correlations varied between 0.48 and 0.97 (Jones et al., *personal communication*).

The use of individual patient data (IPD) in multivariate meta-analysis should also be considered, especially as IPD is the gold-standard for meta-analysis [27] and it would allow any unavailable within-study correlations to be calculated directly [7]. In practice though, IPD may only be available for a proportion of studies, and so methods for multivariate meta-analysis are required that combine IPD and aggregate data [27,28]. There has also be little consideration of how to assess dissemination bias using a multivariate meta-analysis framework [29,30], and this warrants attention as meta-analysis datasets are often fraught with such issues as publication bias [31] and within-study selective reporting [32,33]. In such scenarios some of the missing endpoints may not be missing at random, and so sensitivity analyses to assess how the meta-analysis results change under a variety of missing data assumptions would be potentially valuable [34].

## Conclusion

In this paper we have used analytic reasoning, two applied examples and a realistic simulation study to highlight the benefits of a normal model for BRMA over two separate URMAs, and explain why the between-study correlation is often estimated as +1 or -1. For meta-analysis of proportions, we also extended our work to a generalised model for BRMA, to ensure the binary data is modelled correctly. Our work adds to a growing body of literature indicating the rationale and benefits of a multivariate approach to meta-analysis, and we encourage meta-analysts to consider the approach in practice.

## Abbreviations

BRMA – Bivariate random-effects meta-analysis

IPD – Individual patient data

URMA – Univariate random-effects meta-analysis

REML – Restricted maximum likelihood

## Competing interests

The author(s) declare that they have no competing interests.

## Authors' contributions

RDR, KRA, AJS and PCL developed the study. RDR performed analysis of the telomerase and CD4 data. RDR, PL and JRT performed the normal model simulations; RDR performed the generalized model simulations. All authors examined the simulation results and discussed their interpretations. RDR wrote the paper with contributions from all authors.

## Pre-publication history

The pre-publication history for this paper can be accessed here:



## Supplementary Material

Additional file 1 Appendix 1. Simulation results of the normal BRMA and URMA models for scenarios (iii) to (vii), (x) and (xi) Click here for file

Additional file 2 Appendix 2. Simulation results for the normal BRMA and URMA models for some scenarios involving negative correlation Click here for file

## References

[B1] Berkey CS, Hoaglin DC, Antczak-Bouckoms A, Mosteller F, Colditz GA (1998). Meta-analysis of multiple outcomes by regression with random effects. Stat Med.

[B2] Hasselblad V (1998). Meta-analysis of multitreatment studies. Med Decis Making.

[B3] Becker BJ, Tinsley HEA, Brown S (2000). Multivariate Meta-analysis.

[B4] Van Houwelingen HC, Arends LR, Stijnen T (2002). Advanced methods in meta-analysis: multivariate approach and meta-regression. Stat Med.

[B5] Reitsma JB, Glas AS, Rutjes AW, Scholten RJ, Bossuyt PM, Zwinderman AH (2005). Bivariate analysis of sensitivity and specificity produces informative summary measures in diagnostic reviews. J Clin Epidemiol.

[B6] Thompson JR, Minelli C, Abrams KR, Tobin MD, Riley RD (2005). Meta-analysis of genetic studies using Mendelian randomization--a multivariate approach. Stat Med.

[B7] Daniels MJ, Hughes MD (1997). Meta-analysis for the evaluation of potential surrogate markers. Stat Med.

[B8] Gail MH, Pfeiffer R, Van Houwelingen HC, Carroll RJ (2000). On meta-analytic assessment of surrogate outcomes. Biostatistics.

[B9] Kalaian HA, Raudenbush SW (1996). A Multivariate Mixed Linear Model for Meta-Analysis. Psychological Methods.

[B10] Riley RD, Abrams KR, Lambert PC, Sutton AJ, Thompson JR (2007). An evaluation of bivariate random-effects meta-analysis for the joint synthesis of two correlated outcomes. Statistics in Medicine.

[B11] Arends LR, Voko Z, Stijnen T (2003). Combining multiple outcome measures in a meta-analysis: an application. Stat Med.

[B12] Chu H, Cole SR (2006). Bivariate meta-analysis for sensitivity and specificity with sparse data: a generalized linear mixed model approach (letter to the Editor). Journal of Clinical Epidemiology.

[B13] Glas AS, Roos D, Deutekom M, Zwinderman AH, Bossuyt PM, Kurth KH (2003). Tumor markers in the diagnosis of primary bladder cancer. A systematic review. J Urol.

[B14] Hardy RJ, Thompson SG (1996). A likelihood approach to meta-analysis with random effects. Stat Med.

[B15] Nam IS, Mengersen K, Garthwaite P (2003). Multivariate meta-analysis. Stat Med.

[B16] Berrington A, Cox DR (2003). Generalized least squares for the synthesis of correlated information. Biostatistics.

[B17] Raudenbush SW, Becker BJ, Kalaian H (1988). Modeling multivariate effect sizes. Psychological Bulletin.

[B18] Gentle JE, Gentle JE (1998). Cholesky Factorization. Numerical Linear Algebra for Applications in Statistics.

[B19] Sohn SY (2000). Multivariate meta-analysis with potentially correlated marketing study results. Naval Research Logistics.

[B20] Follmann DA, Proschan MA (1999). Valid inference in random effects meta-analysis. Biometrics.

[B21] Harbord RM, Deeks JJ, Egger M, Whiting P, Sterne JA (2006). A unification of models for meta-analysis of diagnostic accuracy studies. Biostatistics.

[B22] Becker BJ, Hedges LV, Pigott TD (2004). Campbell Collaboration Statistical Analysis Policy Brief. A Campbell Collaboration resource document (available at http://wwwcampbellcollaborationorg/ECG/policy_statasp).

[B23] Westwood ME, Whiting PF, Kleijnen J (2005). How does study quality affect the results of a diagnostic meta-analysis?. BMC Med Res Methodol.

[B24] Sidik K, Jonkman JN A comparison of heterogeneity variance estimators in combining results of studies. Stat Med (in press).

[B25] Abrams KR, Sutton AJ, Cooper NJ, Sculpher MPS, Ginlley L, Robinson M (2005). Populating economic decision models using meta-analyses of heterogenously reported studies augmented with expert beliefs. Technical Report 05-06, Centre for Biostatistics and Genetic Epidemiology, University of Leicester.

[B26] Korn EL, Albert PS, McShane LM (2005). Assessing surrogates as trial endpoints using mixed models. Stat Med.

[B27] Riley RD, Look MP, Simmonds MC (2007). Combining individual patient data and aggregate data in evidence synthesis: a systematic review identified current practice and possible methods. Journal of Clinical Epidemiology (in press).

[B28] Goldstein H, Yang M, Omar RZ, Turner RM, Thompson SG (2000). Meta-analysis using multilevel models with an application to the study of class size effects. Applied Statistics.

[B29] Riley RD, Sutton AJ, Abrams KR, Lambert PC (2004). Sensitivity analyses allowed more appropriate and reliable meta-analysis conclusions for multiple outcomes when missing data was present. Journal of Clinical Epidemiology.

[B30] Jackson D, Copas J, Sutton AJ (2005). Modelling reporting bias: the operative mortality rate for ruptured abdominal aortic aneurysm repair. Journal of the Royal Statistical Society, Series A.

[B31] Sterne JA, Egger M, Smith GD (2001). Systematic reviews in health care: Investigating and dealing with publication and other biases in meta-analysis. BMJ.

[B32] Hahn S, Williamson PR, Hutton JL, Garner P, Flynn EV (2000). Assessing the potential for bias in meta-analysis due to selective reporting of subgroup analyses within studies. Stat Med.

[B33] Hutton JL, Williamson PR (2000). Bias in meta-analysis due to outcome variable selection within studies. Appl Stat.

[B34] Copas J, Shi JQ (2000). Meta-analysis, funnel plots and sensitivity analysis. Biostatistics.

