# Supplementary material for: Bivariate random-effects meta-analysis and the estimation of between-study correlation
Source: BMC Med Res Methodol. 2007 Jan 12;7:3. doi: 10.1186/1471-2288-7-3 (PMC1800862; doi:10.1186/1471-2288-7-3)
Supplement: Additional file 1 — Appendix 1. Simulation results of the normal BRMA and URMA models for scenarios (iii) to (vii), (x) and (xi) [file 1471-2288-7-3-S1.doc]

| **Meta-analysis model** | ***n*** | **No. of**  **the 1000 simulations that**  **converged** | **Bias of mean** $\hat{\beta}_{1}$ | **Mean**  **s.e.**  **of** | **MSE**  **of** | **Coverage**  **of the**  **95% CIs**  **for** $\hat{\beta}_{1}$ | **Bias of mean** | | **Mean**  **s.e.**  **of** | **MSE**  **of** | | **Coverage**  **of the 95% CIs**  **for**$\hat{\beta}_{2}$ | **Bias of mean** $\hat{\tau}_{1}^{2}$ **(no. of $\hat{\tau}_{1}^{2}$= 0)** | **Bias of**  **mean** $\hat{\tau}_{2}^{2}$ **(no. of**  $\hat{\tau}_{2}^{2}$**= 0)** | **Bias of mean** $\hat{\rho}_{B}$ | **% of**  **=**  **-1** | **% of** $\hat{\rho}_{B}$   **=**  **1** |
| --- | --- | --- | --- | --- | --- | --- | --- | --- | --- | --- | --- | --- | --- | --- | --- | --- | --- |
| **Scenario (iii): Complete data - high within-study correlation but no between-study correlation; within-study variation similar to between-study variation** | | | | | | | | | | | | | | | | | |
| URMA | 50 | 1000 | 0.002 | 0.101 | 0.0110 | 94.1% | 0.003 | | 0.106 | 0.011 | | 95.1% | -0.005 (0) | -0.004 (1) | - | - | - |
| BRMA | 50 | 1000 | 0.003 | 0.099 | 0.0106 | 93.7% | 0.003 | | 0.102 | 0.011 | | 94.8% | -0.005 (0) | -0.005 (0) | -0.080 | 2.3% | 0% |
| URMA | 5 | 999 | 0.004 | 0.268 | 0.0814 | 96.2% | 0.004 | | 0.269 | 0.080 | | 95.5% | -0.004 (109) | 0.019 (78) | - | - | - |
| BRMA | 5 | 997 | 0.006 | 0.272 | 0.0826 | 97.1% | 0.004 | | 0.272 | 0.083 | | 95.4% | 0.019 (6) | 0.037 (0) | -0.073 | 35.9% | 17.5% |
| **Scenario (iv):Complete data - high within- and between-study correlation; within-study variation similar to between-study variation** | | | | | | | | | | | | | | | | | |
| URMA | 50 | 1000 | -0.004 | 0.102 | 0.0102 | 94.5% | -0.007 | | 0.106 | 0.011 | | 94.6% | -0.002 (0) | -0.003 (0) | - | - | - |
| BRMA | 50 | 1000 | -0.003 | 0.095 | 0.0089 | 95.1% | -0.005 | | 0.097 | 0.009 | | 93.9% | 0 (0) | -0.002 (0) | -0.014 | 0% | 2.0% |
| URMA | 5 | 1000 | -0.008 | 0.271 | 0.0834 | 97.9% | -0.016 | | 0.262 | 0.092 | | 93.2% | 0.004 (84) | 0.001 (106) | - | - | - |
| BRMA | 5 | 998 | -0.009 | 0.262 | 0.0805 | 97.7% | -0.016 | | 0.254 | 0.087 | | 92.9% | 0.012 (8) | 0.009 (0) | -0.149 | 12.4% | 53.6% |
| **Scenario (v)*: Complete data - high within- and between-study correlation; within-study variation large relative to between-study variation** | | | | | | | | | | | | | | | | | |
| URMA | 50 | 998 | 0.001 | 0.046 | 0.00243 | 93.2% | 0.003 | 0.067 | | | 0.004 | 96.4% | 0.005 (229) | 0.011 (143) | - | - | - |
| BRMA | 50 | 951 | 0.001 | 0.036 | 0.00157 | 90.1% | 0.002 | 0.048 | | | 0.002 | 94.7% | 0.003 (74) | 0.005 (0) | -0.441 | 21.9% | 56.2% |
| **Scenario (vi)*: Complete data - high within- and between-study correlation; within-study variation large (endpoint 1) and small (endpoint 2) relative to between-study variation** | | | | | | | | | | | | | | | | | |
| URMA | 50 | 1000 | -0.001 | 0.046 | 0.0024 | 93.4% | 0.004 | | 0.199 | 0.041 | | 94.7% | 0.005 (271) | -0.005 (0) | - | - | - |
| BRMA | 50 | 980 | -0.002 | 0.040 | 0.0021 | 90.5% | 0.001 | | 0.192 | 0.039 | | 94.7% | 0.007 (15) | 0.011 (0) | -0.336 | 10.8% | 52.2% |
| **Scenario (vii): Complete data - high within- and between-study correlation; within-study variation small relative to between-study variation** | | | | | | | | | | | | | | | | | |
| URMA | 50 | 1000 | -0.001 | 0.198 | 0.038 | 95.8% | -0.005 | | 0.200 | 0.038 | | 94.9% | 0.015 (0) | 0.010 (0) | - | - | - |
| BRMA | 50 | 1000 | 0.001 | 0.194 | 0.036 | 95.7% | -0.005 | | 0.196 | 0.036 | | 95.6% | 0.009 (0) | 0.014 (0) | -0.002 | 0% | 0% |
| URMA | 5 | 1000 | 0.019 | 0.542 | 0.335 | 94.5% | 0.011 | | 0.552 | 0.346 | | 94.1% | 0.012 (10) | 0.025 (6) | - | - | - |
| BRMA | 5 | 1000 | 0.018 | 0.538 | 0.335 | 94.6% | 0.012 | | 0.546 | 0.348 | | 94.1% | 0.011 (0) | 0.022 (0) | -0.055 | 23.0% | 19.0% |
| **Scenario (x): Missing data - no within-study correlation, but high between-study correlation; within-study variation small relative to between-study variation** | | | | | | | | | | | | | | | | | |
| URMA | 50 | 1000 | -0.004 | 0.0708 | 0.005 | 94.9% | 0 | | 0.099 | 0.010 | | 95.0% | -0.006 (0) | -0.005 (0) | - | - | - |
| BRMA | 50 | 1000 | -0.004 | 0.0708 | 0.005 | 94.9% | 0 | | 0.082 | 0.007 | | 95.2% | -0.006 (0) | -0.007 (0) | -0.001 | 0% | 0% |
| URMA | 10 | 1000 | -0.002 | 0.154 | 0.028 | 94.1% | -0.003 | | 0.209 | 0.058 | | 93.7% | -0.006 (0) | -0.006 (0) | - | - | - |
| BRMA | 10 | 1000 | -0.002 | 0.154 | 0.028 | 94.1% | -0.001 | | 0.174 | 0.043 | | 93.3% | -0.006 (0) | 0.006 (0) | -0.040 | 0% | 3.9% |
| **Scenario (xi): Missing data - no within-study correlation, but high between-study correlation; within-study variation similar to between-study variation** | | | | | | | | | | | | | | | | | |
| URMA | 50 | 1000 | -0.005 | 0.102 | 0.010 | 95.6% | -0.001 | | 0.145 | 0.023 | | 94.2% | 0 (0) | -0.003 (6) | - | - | - |
| BRMA | 50 | 1000 | -0.004 | 0.101 | 0.010 | 95.8% | -0.003 | | 0.137 | 0.020 | | 94.7% | 0.001 (0) | 0.003 (0) | -0.012 | 0.1% | 35.6% |
| URMA | 10 | 1000 | -0.001 | 0.218 | 0.045 | 93.9% | -0.005 | | 0.263 | 0.083 | | 94.2% | 0.006 (45) | 0.005 (84) | - | - | - |
| BRMA | 10 | 997 | -0.001 | 0.222 | 0.045 | 96.5% | -0.007 | | 0.255 | 0.080 | | 95.6% | 0.020 (0) | 0.025 (0) | -0.164 | 10.1% | 60.2% |

MSE = mean-square-error, *n* = number of studies in each meta-analysis, CIs = confidence intervals, s.e. = standard error;

* For settings (v) and (vi) the *n = 5* simulations were not possible due to problems of non-convergence
