# Supplementary material for: Bivariate random-effects meta-analysis and the estimation of between-study correlation
Source: BMC Med Res Methodol. 2007 Jan 12;7:3. doi: 10.1186/1471-2288-7-3 (PMC1800862; doi:10.1186/1471-2288-7-3)
Supplement: Additional file 2 — Appendix 2. Simulation results for the normal BRMA and URMA models for some scenarios involving negative correlation [file 1471-2288-7-3-S2.doc]

| **Meta-analysis model** | ***n*** | **No. of**  **the 1000 simulations that**  **converged** | **Bias of mean** $\hat{\beta}_{1}$ | **Mean**  **s.e.**  **of** | **MSE**  **of** | **Coverage**  **of the**  **95% CIs**  **for** $\hat{\beta}_{1}$ | **Bias of mean** | **Mean**  **s.e.**  **of** | **MSE**  **of** | **Coverage**  **of the 95% CIs**  **for**$\hat{\beta}_{2}$ | **Bias of mean** $\hat{\tau}_{1}^{2}$ **(no. of $\hat{\tau}_{1}^{2}$= 0)** | **Bias of**  **mean** $\hat{\tau}_{2}^{2}$ **(no. of**  $\hat{\tau}_{2}^{2}$**= 0)** | **Bias of mean** $\hat{\rho}_{B}$ | **% of**  **=**  **-1** | **% of** $\hat{\rho}_{B}$   **=**  **1** |
| --- | --- | --- | --- | --- | --- | --- | --- | --- | --- | --- | --- | --- | --- | --- | --- |
| **Complete data as for scenario (ii) except the between-study correlation was -0.8** | | | | | | | | | | | | | | | |
| URMA | 5 | 1000 | 0.006 | 0.267 | 0.080 | 96.4% | 2.008 | 0.266 | 0.0888 | 93.7% | -0.004 (89) | 0.013 (87) | - | - | - |
| BRMA | 5 | 998 | 0.005 | 0.269 | 0.079 | 98.7% | 2.012 | 0.283 | 0.0927 | 96.4% | 0.017 (5) | 0.027 (0) | 0.053 | 20.4% | 34.5% |
| **Complete data as for scenario (ii) except the within-study correlations were -0.8 in 25 studies & 0.8 in others** | | | | | | | | | | | | | | | |
| URMA | 50 | 1000 | -0.003 | 0.102 | 0.0103 | 95.4% | 0 | 0.105 | 0.0114 | 94.9% | -0.003 (0) | -0.013 (1) | - | - | - |
| BRMA | 50 | 1000 | -0.003 | 0.091 | 0.0079 | 95.1% | 0.001 | 0.092 | 0.0088 | 95.7% | -0.001 (0) | -0.009 (0) | 0 | 0% | 8.6% |
| **Missing data as for scenario (xi) except the within- and between-study correlations were all -0.8** | | | | | | | | | | | | | | | |
| URMA | 50 | 1000 | -0.007 | 0.102 | 0.011 | 93.8% | 0.010 | 0.146 | 0.023 | 93.7% | 0 (0) | 0.004 (0) | - | - | - |
| BRMA | 50 | 1000 | -0.008 | 0.099 | 0.011 | 94.1% | 0.009 | 0.119 | 0.017 | 94.7% | 0.002 (0) | 0.003 (0) | 0.010 | 88.0% | 2.0% |

MSE = mean-square-error, *n* = number of studies in each meta-analysis, CIs = confidence intervals, s.e. = standard error
